# NapA Mediates a Redox Regulation of the Antioxidant Response, Carbon Utilization and Development in *Aspergillus nidulans*

**DOI:** 10.3389/fmicb.2017.00516

**Published:** 2017-03-30

**Authors:** Ariann E. Mendoza-Martínez, Fernando Lara-Rojas, Olivia Sánchez, Jesús Aguirre

**Affiliations:** Departamento de Biología Celular y del Desarrollo, Instituto de Fisiología Celular, Universidad Nacional Autónoma de MéxicoCoyoacán, Mexico

**Keywords:** ROS, cleistothecia, secondary metabolism, iron scavenging, germination

## Abstract

The redox-regulated transcription factors (TFs) of the bZIP AP1 family, such as yeast Yap1 and fission yeast Pap1, are activated by peroxiredoxin proteins (Prxs) to regulate the antioxidant response. Previously, *Aspergillus nidulans* mutants lacking the Yap1 ortholog NapA have been characterized as sensitive to H_2_O_2_ and menadione. Here we study NapA roles in relation to TFs SrrA and AtfA, also involved in oxidant detoxification, showing that these TFs play different roles in oxidative stress resistance, catalase gene regulation and development, during *A. nidulans* life cycle. We also uncover novel NapA roles in repression of sexual development, normal conidiation, conidial mRNA accumulation, and carbon utilization. The phenotypic characterization of Δ*gpxA*, Δ*tpxA*, and Δ*tpxB* single, double and triple peroxiredoxin mutants in wild type or Δ*napA* backgrounds shows that none of these Prxs is required for NapA function in H_2_O_2_ and menadione resistance. However, these Prxs participate in a minor NapA-independent H_2_O_2_ resistance pathway and NapA and TpxA appear to regulate conidiation along the same route. Using transcriptomic analysis we show that during conidial development NapA-dependent gene expression pattern is different from canonical oxidative stress patterns. In the course of conidiation, NapA is required for regulation of at least 214 genes, including ethanol utilization genes *alcR, alcA* and *aldA*, and large sets of genes encoding proteins involved in transcriptional regulation, drug detoxification, carbohydrate utilization and secondary metabolism, comprising multiple oxidoreductases, membrane transporters and hydrolases. In agreement with this, Δ*napA* mutants fail to grow or grow very poorly in ethanol, arabinose or fructose as sole carbon sources. Moreover, we show that NapA nuclear localization is induced not only by oxidative stress but also by growth in ethanol and by carbon starvation. Together with our previous work, these results show that SakA-AtfA, SrrA and NapA oxidative stress-sensing pathways regulate essential aspects of spore physiology (i.e., cell cycle arrest, dormancy, drug production and detoxification, and carbohydrate utilization).

## Introduction

The proposed role of reactive oxygen species (ROS) as essential cell differentiation signals (Hansberg and Aguirre, 1990; Aguirre et al., 2005) led us to study the mechanisms by which eukaryotic cells detoxify ROS, using the model fungus *Aspergillus nidulans*. To perceive and transmit oxidative stress signals, fungi utilize phosphorelay systems connected to MAP kinases specialized in transducing stress signals or SAPKs. *Schizosaccharomyces pombe* paradigmatic SAPK Sty1/Spc1 has been characterized as a MAPK involved in cell-cycle control (Shiozaki and Russell, 1995) that is activated by osmotic (Millar et al., 1995; Degols et al., 1996), oxidative (Degols et al., 1996), heat shock (Nguyen and Shiozaki, 1999), nitrogen limitation (Shiozaki and Russell, 1996), and UV light (Degols and Russell, 1997) stress. As indicated in Figure S1 and Table S1, the phosphorelay system linked to Sty1/Spc1 is composed by histidine kinases (HK) Mak1, Mak2, and Mak3 (Buck et al., 2001), the phosphotransfer protein (HPt) Mpr1 and the response regulator (RR) Mcs4. Sty1/Spc1 in turn regulates transcription factor Atf1. Despite the architecture similarity to *Saccharomyces cerevisiae* Sln1-Ypd1-Ssk1-Hog1 system (de Nadal et al., 2011), *S*. *pombe* phosphorelay transmits oxidative, not osmotic stress signals (Nguyen et al., 2000). A second phosphorelay component, the transcription factor Prr1 is also required for oxidative stress responses, independently of Sty1/Spc1 (Quinn et al., 2011). In addition to Atf1 and Prr1, transcription factor Pap1, a homolog of *S. cerevisiae* Yap1 (Moye-Rowley et al., 1989), is critical for the antioxidant response in this fungus. The oxidation signal is perceived by different peroxiredoxins or Prxs and then transmitted to Pap1 or Yap1, which once oxidized accumulate in the nucleus to regulate the expression of multiple genes involved in the antioxidant response.

All peroxiredoxins belong to a conserved family of peroxidases that reduce peroxide and contain a conserved “peroxidatic” cysteine. Peroxides oxidize this Cys to sulphenic acid, which then reacts with another “resolving” Cys to form a disulfide bond, subsequently reduced by a suitable electron donor to complete a catalytic cycle. Prxs are classified into 2-Cys, atypical 2-Cys and 1-Cys families. 2-Cys are homodimeric and contain peroxidatic and resolving Cys residues in the same subunit. However, the disulfide bond is formed between two different subunits. In atypical 2-Cys an intermolecular disulfide is formed within the same subunit. 1-Cys Prxs form a disulfide with a resolving Cys present in other proteins or small thiol molecules (Rhee, 2016). Until now, typical 2-Cys Prxs have not been found in filamentous fungi. The role of *S. cerevisiae* peroxiredoxin Gpx3 in Yap1 activation, which also requires Yap1-binding protein Ybp1, was the first description of Prx function in H_2_O_2_ sensing (Delaunay et al., 2002). However, under certain conditions peroxiredoxin Tsa1 can also mediate Yap1 activation by H_2_O_2_ (Tachibana et al., 2009). In *S. pombe*, the 2-Cys peroxiredoxin Tpx1 transmits the redox signal to Pap1 (Vivancos et al., 2004, 2005).

*A. nidulans* contains 15 HKs and the function of most of them is unknown. Genetic evidence indicates that HK NikA transmits osmostress and fungicide signals to (HPt) YpdA and to SrkA RR, which is coupled to the SAPK SakA/HogA (Han and Prade, 2002; Kawasaki et al., 2002), as well as to the SAPK-independent RR SrrA (Hagiwara et al., 2007; Vargas-Perez et al., 2007). Upstream MAPKK PbsB and MAPKKK SskB regulate SakA (Furukawa et al., 2005), which is able to replace Sty1/Spc1 functions in *S. pombe*, and in *A. nidulans* is phosphorylated in response to multiple types of stress, including osmotic, oxidative (Kawasaki et al., 2002), nutrient starvation (Lara-Rojas et al., 2011) and hypoxia (Sánchez and Aguirre, unpublished). Stress-activated SakA translocates to the nucleus, where it interacts with transcription factor AtfA, required for induction of multiple genes and both, Δ*sakA* and Δ*atfA* mutants are sensitive to oxidative stress (Lara-Rojas et al., 2011). Additionally, SakA and AtfA are required for osmotic-induced gene expression (Hagiwara et al., 2009).

TF SrrA is also needed for oxidative stress resistance (Vargas-Perez et al., 2007) and both, SakA and SrrA play important roles during development. SakA represses sexual development and is activated during asexual development (Kawasaki et al., 2002). Δ*sakA* intact conidia progressively lose their viability and this is consistent with the fact that phosphorylated SakA accumulates in asexual spores (conidia) in an AtfA-dependent manner, and its dephosphorylation is necessary for germination to take place (Lara-Rojas et al., 2011). Likewise, Δ*srrA* mutants show severely decreased asexual sporulation and produce conidia that very rapidly lose their viability (Vargas-Perez et al., 2007). In addition to transcription factors (TFs) AtfA and SrrA, the Yap1/Pap1 functional homolog NapA has been shown to be required for resistance to H_2_O_2_ in *A. nidulans* (Asano et al., 2007). Unrelated protein AN8863, putatively involved in nucleosome assembly, was later also referred to as NapA (Araújo-Bazan et al., 2008). Here we keep using NapA to name the *A. nidulans* Yap1/Pap1 homolog (AN7513) because it has been used this way in other publications (Lessing et al., 2007; Thön et al., 2010), and because the name “ap” preceded by the first letter of the species name (i.e., *n**idulans* apA) has been widely used in many other filamentous fungi, where the role of Yap1/Pap1 homologs in oxidative stress resistance has been demonstrated (Lessing et al., 2007; Qiao et al., 2008; Temme and Tudzynski, 2009; Tian et al., 2011; Cartwright and Scott, 2013). Notably, in several plant pathogens Yap1/Pap1 homologs are involved not only in regulation of the antioxidant response but also in plant virulence (Molina and Kahmann, 2007; Guo et al., 2011; Huang et al., 2011).

Here we compared the relative contribution of (TFs) AtfA, SrrA and NapA to the antioxidant response and development in *A. nidulans* and uncovered novel NapA roles in regulation of sexual and asexual development, carbon utilization and gene regulation during asexual sporulation.

## Materials and methods

### Strains, media, growth conditions, and catalase activity determination

The *A. nidulans* strains used in this work are listed in Table S2. All strains were grown at 37°C in glucose minimal (MM) nitrate medium (Hill and Käfer, 2001), plus supplements. Δ*napA* strains in a *veA*^+^ background were obtained from sexual crosses with strain FGSCA4. The presence of wild type *veA* allele was confirmed by PCR using genomic DNA from selected progeny and the primers veAforward and veAreverse, as reported (Han et al., 2010). Menadione was filter sterilized and like H_2_O_2_, added to agar medium at 50°C before solidification. H_2_O_2_-containing plates were used the day they were prepared or stored at 4°C for no more than 24 h. Since H_2_O_2_ can react with medium components, the actual concentration in plates cannot be estimated. To ensure experimental reproducibility, the same batch of H_2_O_2_ containing medium was used when comparing different strains. Spore suspensions containing 1 × 10^3^ or 1 × 10^4^ conidia were used to inoculate plates by dropping the suspension on the center of plates containing different stressors or media. Higher H_2_O_2_ resistance is observed at higher spore densities, presumably due to the high catalase A activity levels found in conidia (Navarro et al., 1996; Navarro and Aguirre, 1998). For catalase activity, 30 μg of total protein extracts prepared from conidia or mycelia were separated on native polyacrylamide gels to determine catalase activity as reported (Navarro et al., 1996; Kawasaki et al., 1997). Briefly gels are incubated in 5% methanol with shaking for 5 min and then rinsed with tap water 3 times. After this, the gel is incubated in a 0.03% hydrogen peroxide solution (100 μl of commercial 30% solution in 100 ml of deionized water) for 5 min and rinsed with water. Finally, the gel is incubated in the staining solution until the bands of activity are visible. The staining solution is made by mixing equal volumes of a 2% (w/v) FeCl_3_ solution and a 2% (w/v) K_3_Fe(CN)_6_. The different catalases were mapped before using single, double and triple mutants affected in *catA, catB*, and *catC* genes (Kawasaki and Aguirre, 2001).

### Deletion of *napA, gpxA, tpxA, tpxB* and *alcA* genes, and tagging of NapA

Genomic DNA was used as template to produce the gene-deletion constructs by double joint PCR (Yu et al., 2004). For *napA* gene (AN7513) replacement construct, the *napA* ORF was amplified with primers 5′ For-napA and 5′ Rev-napA (see Table S3). The 3′ *napA* fragment was amplified with primers 3′ For-*napA* and 3′ Rev-*napA. Aspergillus fumigatus pyrG* marker was amplified with primers pyrGforward and pyrGreverse, using plasmid PFNO3 as template (Nayak et al., 2006). The three fragments were purified, mixed and used in a fusion PCR with primers 5′nest-napA and 3′nest-napA. The final 4900 bp napA–AfpyrG–napA cassette was purified and used to transform *A. nidulans* strain 11035 by electroporation (Sanchez and Aguirre, 1996; Sánchez et al., 1998). Five PyrG^+^ transformants were obtained and analyzed by Southern blot to confirm the elimination of *napA*. After confirming the proper deletion event (Figure S2), strain TFL9 was chosen and crossed with strain CLK43 to get rid of the *kuA* deletion, and progeny strain CFL7 was confirmed by PCR and used in further experiments.

To delete the *gpxA* gene (AN2846), primer pairs 5′For-*gpxA*/5′Rev-*gpxA* and 3′For-*gpxA*/3′Rev-*gpxA* were used to amplify *gpxA* 5′ and 3′ regions, respectively. Primers 5′For-nested *gpxA* and 3′Rev-nested *gpxA* were used to obtain the final fusion product. *A. fumigatus riboB* marker was amplified with primers 5Ribo and 6Ribo, using plasmid pAfriboPstE1Skt(ssp1)-37 as template (Nayak et al., 2006). The 5000 bp *gpxA*-AfriboB-*gpxA* cassette was purified as before and used to transform *A. nidulans* strain 11035 by electroporation. Twenty RiboB^+^ transformants were obtained, and analyzed by PCR to confirm the elimination of *gpxA* (Figure S3). Six transformants contained the expected event. Strain TAM16 was crossed with strain CLK43 and progeny strain CAM11 was confirmed by PCR and used in further experiments.

A similar strategy was used to delete the *tpxA* gene (AN10223). Primers pairs 5′For-*tpxA*/5′Rev-*tpxA* and 3′For-*tpxA*/3′Rev-*tpxA* were used to amplify *tpxA* 5′ and 3′ regions, respectively. *A. fumigatus pyrG* marker was amplified with primers pyrGforward and pyrGreverse, using plasmid PFNO3 as template (Nayak et al., 2006). The 3483 bp fusion *tpxA*-AfpyrG-*tpxA* PCR product obtained with primers 5′nest tpxA and 3′nest tpxA was used to transform strain 11035 by electroporation. 15 transformants obtained were analyzed by PCR, and 4 transformants were confirmed (Figure S3). Transformant 6 was named TAM17, crossed with strain CLK43 and progeny strain CAM13 confirmed by PCR and chosen for additional experiments.

To delete *tpxB* gene (AN3973), primer pairs 5′For-*tpxB*/5′Rev-*tpxB* and 3′For-*tpxB*/3′Rev-*tpxB* were used to amplify *tpxB* 5′ and 3′ regions, respectively. The *A. fumigatus pyrG* marker was amplified as before and the 3483 bp fusion PCR product obtained with primers 5′nest tpxB and 3′nest tpxB was used to transform strain 11035 by electroporation. 4 transformants out of 5 obtained were confirmed by PCR (Figure S3). Transformant 4 was named TAM19, crossed with strain CLK43 and progeny strain CAM19 used in further experiments.

The *alcA* deletion construct containing the *riboB* gene, as a selective marker, was generated using primers 5′For-*alcA* and 5′Rev-*alcA* for 5′ region and 3′For-*alcA* and 3′Rev-*alcA* for the 3′ region. *A. fumigatus riboB* marker was amplified with primers 5Ribo and 6Ribo as before. The 4167 bp band obtained with primers NestForalcA and NestRevaclA was used to transform strain 11035 by electroporation. Six transformants contained the expected event (Figure S4). Transformant TAM20 was crossed with strain CLK43 and progeny strain CAM17 was confirmed by PCR and chosen for further experiments. Sexual crosses generated double, triple and quadruple mutants, as indicated in Table S2.

To generate NapA::GFP C-terminal construct, three PCR products were used. First, 5′ and entire *napA* ORF were amplified with primers GSP1napA and GSP2napA. Second, a 3′ *napA* fragment was amplified with primers GSP3napA and GSP4napA. Third, GFP and *A. fumigatus pyrG* marker were amplified with primers GFP1napA and GFP2napA as before. Purified fragments were used in a fusion PCR with primers GSP1napA and GSP4napA. The 6625 bp napA–GFP–AfpyrG cassette was used to transform *A. nidulans* strain A1155 by electroporation. Transformant TFL14 strain was confirmed by PCR and used for further experiments (Figure S5).

### Microscopy

Fluorescence microscopy images were captured *in vivo*. Spores from NapA::GFP strain were grown for 18 h on coverslips containing liquid glucose-MM at 37°C with no shaking. After this, samples were shifted to the same medium with or without 2 mM H_2_O_2_, incubated for 0–120 min and observed using a NIKON Eclipse E600 microscope to detect DAPI and GFP fluorescence. Images were captured with a cooled camera Neo Andor sCMOS. For DAPI staining, samples of conidia or mycelia were fixed in methanol/acetone for 10 min, washed in water and stained for 5 min with 0.1 mg/ml of 4′,6-diamidino-2-phenylindole (DAPI).

### Transcriptomic analysis

Conidia from 6-day old cultures from strains CLK43 (WT) and CFL7 (Δ*napA*) were collected and washed 3 times with 10 ml of cold water. Excess liquid was removed by centrifugation and conidia were immediately frozen with liquid nitrogen. Total RNA was extracted by cryogenic grinding using the Tissue Lyser (Qiagen) and purified using the RNAeasy Mini kit (Qiagen), following the manufacturer's protocol. RNA integrity number (RIN) for RNA quality was generated using the Agilent 2100 Bioanalyzer System (Agilent technologies). Two independent samples from each strain (Biological replicates) were processed for cDNA synthesis using Illumina's kit TrueSeqV2 and sequenced at the next-generation sequencing core facility at IBT-UNAM. using Illumina's platform. An average of 10168 reads of 72 bp per sample were obtained, representing nearly 96.3% *A. nidulans* genome lengths. Biological replicates showed a good level of correlation (r_0.966 for WT and r_0.968 for Δ*napA*). Differential gene expression was inferred based on total mapping counts using the EdgeR package. Genes showing a value of log fold change (LFC) ≥2 and a false discovery rate (FDR) ≤ 0.05 were considered as differentially expressed (DE). The differential expression analysis was deposited under the GEO identifier GSE94747, as part of the BioProject PRJNA373914. The sequencing raw reads for all experiments were deposited in the SRA database under the SRP099165 identifier.

### Real-time PCR

Total RNA was isolated using the Plant RNA purification kit (Quiagen) according to the manufacturer's instructions and treated with DNase Turbo DNA-free kit (Ambion). The RNA integrity (RIN) and concentration were determinate using the 2200 TapeStation (Agilent Genomics). 1 μg of RNA was used to synthetize cDNA templates for PCR amplification, using SuperScript III reverse transcriptase (Invitrogen) according to the manufacturer's instructions. The expression of each gene was measured using 1:10 cDNA sample dilutions. Specific primers used for RT-PCR (Table S3) were devised to produce cDNA amplicons around 130 pb. To estimate genomic DNA contamination, primers were designed to amplify a larger product, including an intron of each gene, from a genomic template. RT-PCR products were observed in 2% agarose gels.

Triplicates from each sample were performed. RT-PCR was done using Platinum SYBR Green qPCR SuperMix-UDG with ROX kit (Invitrogen). RT-PCR was performed in a StepOne Real-Time PCR System (Applied Biosystems). The program used included an initial UDG incubation for 2 min at 50°C, followed by a 2 min denaturation step at 95°C, and 40 amplification cycles at 95° C for 15 s, followed by 30 s at 60°C. For relative quantification, we used the Ct comparative method and the data was analyzed with StepOne Software V2.3 (Applied Biosystems), using histone 2B (H2B) gene (AN3469) as reference.

## Results

### TFs NapA, SrrA, and AtfA play differential roles in *A. nidulans* antioxidant response

**Figure 1:**
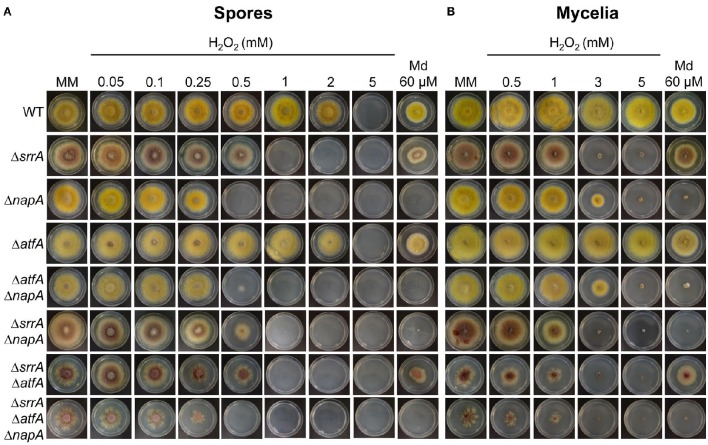
**NapA, SrrA and AtfA play differential roles in *Aspergillus nidulans* antioxidant response. (A)** Conidia (1 × 10^3^) from strains CLK43 (WT), COSsrrA3 (Δ*srrA*), CFL7 (Δ*napA*), TFLΔatfA-04 (Δ*atfA*), CAM7 (Δ*atfA* Δ*napA*), CAM6 (Δ*srrA* Δ*napA*), CAM8 (Δ*srrA* Δ*atfA)*, and CAM9 (Δ*srrA* Δ*atfA* Δ*napA*) were inoculated by dropping spore suspensions on the center of supplemented MM plates containing H_2_O_2_ or menadione (Md) at the indicated concentrations, and incubated at 37°C for 4 days. **(B)** Mycelial plugs cut from the growing edge of 5-day old colonies from strains CLK43, COSsrrA3, CFL7, TFLΔatfA-02, CAM7, CAM6, CAM8, and CAM9 were transferred to plates containing H_2_O_2_ or menadione at the indicated concentrations, and incubated at 37°C for 4 days.

Previous work has shown that (TFs) SrrA (Vargas-Perez et al., 2007), AtfA (Lara-Rojas et al., 2011) and NapA (Asano et al., 2007) are involved in *A. nidulans* antioxidant response. To analyse the relative contribution of each TF in this process, we compared the sensitivity of conidia and mycelia from Δ*napA*, Δ*srrA*, and Δ*atfA* mutants to H_2_O_2_ and menadione. Results show that conidia from Δ*napA* mutant were the most sensitive to H_2_O_2_ followed by Δ*srrA* and Δ*atfA* mutants, while only Δ*napA* mutants were sensitive to menadione (Figure 1A). In contrast, the same assay carried out with mycelia showed a slightly higher H_2_O_2_ sensitivity for Δ*srrA* mutants followed by Δ*napA*, while the *atfA* mutant was as resistant to H_2_O_2_ as the WT strain. Again only the Δ*napA* mutant was sensitive to menadione (Figure 1B).

To further dissect NapA, SrrA, and AtfA contribution to the antioxidant response, we used Δ*napA*, Δ*srrA*, and Δ*atfA* single mutants to perform sexual crosses and obtain double and triple mutants, which were confirmed by PCR analysis (not shown). Results in Figure 1A show that conidia from double and triple mutants did not show additive phenotypes and those carrying the Δ*napA* deletion were as sensitive to H_2_O_2_ and menadione as the single Δ*napA* mutant, except in the case of the Δ*srrA* Δ*napA* strain whose H_2_O_2_ sensitivity was similar to the one displayed by the Δ*srrA* mutant (Figure 1A). In the test performed with mycelia, double and triple mutants carrying the Δ*srrA* deletion behaved as the single Δ*srrA* mutant (Figure 1B). Notably, Δ*srrA* mutant growth defects (Vargas-Perez et al., 2007) are enhanced by the presence of the Δ*atfA* deletion, as colonies from Δ*srrA* Δ*atfA* mutants show a higher decrease in growth and highly irregular colony borders. These growth and conidiation defects were even more drastic in the mutant lacking the 3 TFs (Figures 1A,B).

These results show that TFs NapA, SrrA, and AtfA play different roles at different stages of *A. nidulans* life cycle. For H_2_O_2_ spore resistance NapA plays a more prominent role than SrrA, which in turn is more important than AtfA. For mycelial H_2_O_2_ resistance SrrA is somewhat more critical than NapA, while AtfA plays no role in this process, and only NapA is necessary for menadione resistance in both spores and mycelia. During vegetative growth, neither NapA nor AtfA are individually required for normal radial growth. However, Δ*srrA* mutant growth defects are enhanced by the deletion of AtfA, indicating that AtfA contributes to normal radial growth. SrrA is almost essential for normal conidiation, NapA is needed for full conidiation (see further) and AtfA is dispensable for the process. Regarding conidial function, SrrA and AtfA are critical for conidial viability, and NapA is necessary for the accumulation of multiple mRNAs in conidia (see further).

### NapA, SrrA, and AtfA regulate different catalase genes

**Figure 2:**
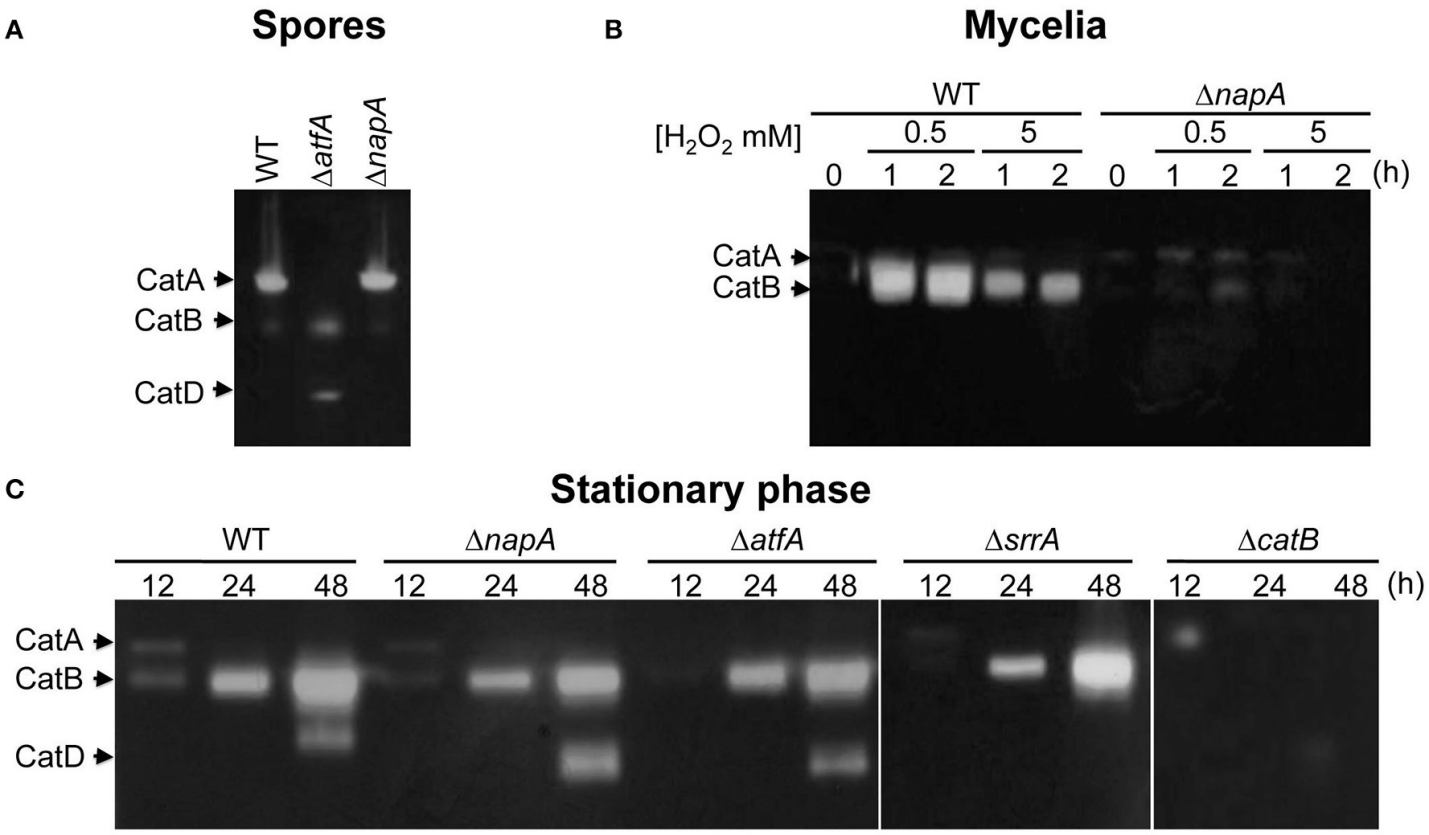
**Transcriptional factors NapA, SrrA and AtfA play differential roles in catalase regulation. (A)** CatA activity levels are not affected in Δ*napA* conidia. Conidial protein extracts from strains CLK43 (WT), TFLΔatfA-04 (Δ*atfA*) and CFL7 (Δ*napA*) were analyzed for catalase in-gel activity. **(B)** CatB activity induction by H_2_O_2_ requires NapA. Mycelia from the strains CLK43 (WT) and CFL7 (Δ*napA*) were grown for 12 h in liquid MM and then H_2_O_2_ was added at the indicated times and concentrations and protein extracts were used for catalase activity determination. **(C)** NapA, AtfA, and SrrA are not required for CatB induction during the stationary phase of growth. Mycelia from the strains CLK43 (WT), CFL7 (Δ*napA*), TFLΔatfA-02 (Δ*atfA*), COSsrrA3 (Δ*srrA*) and TLK12 (Δ*catB*) was grown for 12, 24, and 48 h in liquid MM medium. The experiment was repeated at least 3 times; a representative experiment is shown.

To further understand NapA roles in the antioxidant response, we compared the H_2_O_2_ sensitivity of conidia from mutants lacking NapA, the spore-specific catalase CatA (Navarro et al., 1996; Navarro and Aguirre, 1998) or the mycelium inducible catalase CatB (Kawasaki et al., 1997). Notably, Δ*napA* conidia were much more sensitive to H_2_O_2_ than Δ*catA* conidia, while Δ*catB* conidia showed only a minor sensitivity at 4 mM H_2_O_2_ (Figure S6). As shown in Figure 2A, the presence of CatA activity was completely dependent on AtfA, while being independent of NapA. In contrast, H_2_O_2_ induction of mycelial catalase CatB was largely dependent on both NapA (Figure 2B) and SrrA (Vargas-Perez et al., 2007). Since CatB is also highly induced during the stationary phase of growth (Kawasaki and Aguirre, 2001), we asked if NapA, SrrA or AtfA were required for this induction. Unexpectedly, none of these TFs was needed for this process. However, the induction of catalase-peroxidase CatD/CpeA activity (Kawasaki and Aguirre, 2001; Scherer et al., 2002) required of SrrA (Figure 2C). In summary, these results show that NapA and SrrA are both required for CatB induction by H_2_O_2_, AtfA is required for CatA expression in conidia and none of them is required for CatB induction during the late stationary phase of growth. This and SrrA regulation of CatD/CpeA activity (Kawasaki and Aguirre, 2001) confirm the differential roles that these TFs perform during *A. nidulans* antioxidant response.

### NapA localizes in nuclei in response to oxidative stress

**Figure 3:**
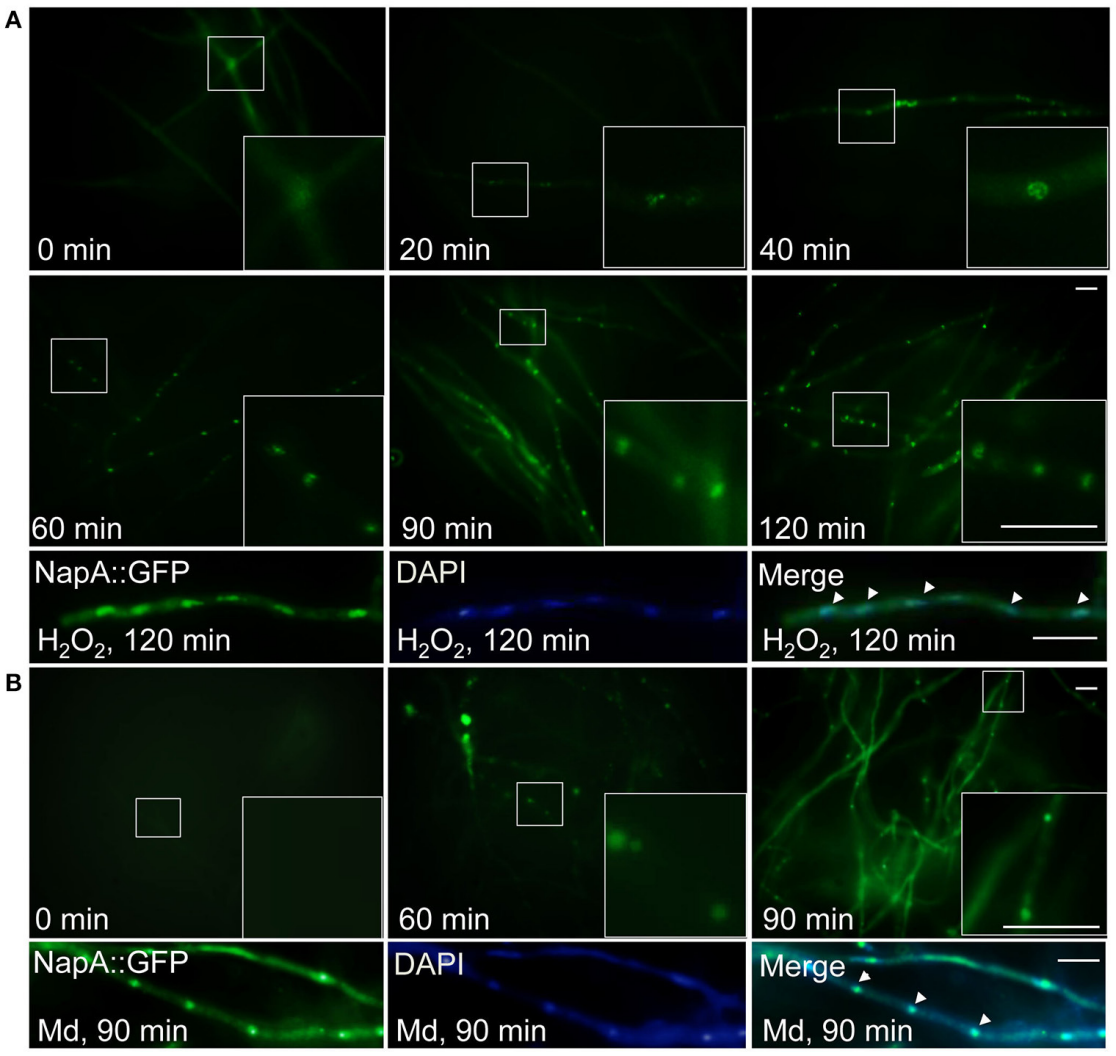
**NapA is induced by oxidative stress and gradually accumulates in nuclei in response to oxidative stress. (A)** NapA nuclear localization increases during incubation with H_2_O_2_. Conidia from strain CAM20 (NapA::GFP) were grown for 18 h in MM and then exposed to 2 mM H_2_O_2_ for the indicated times (0–120 min), observed *in vivo* and photographed every 10 min using Epifluorescence microscopy. Lower panel shows NapA::GFP and nuclei (DAPI) signal in mycelia treated with H_2_O_2_ for 120 min, fixed and photographed. **(B)** Menadione also induces nuclear localization of NapA. Conidia from strain CAM20 (NapA::GFP) were grown for 18 h in MM and then exposed to 10 μM menadione for the indicated times (0–90 min). Larger square areas in each picture show enlargements of the areas indicated by smaller squares. Bars = 10 μm.

The high sensitivity of Δ*napA* conidia to H_2_O_2_, not explained by a simple lack of spore catalase activity (Figure 2A), suggests that NapA is required for other conidial functions, including those involved in resistance to menadione. It is well known that *S. cerevisiae, S. pombe* and other fungal NapA homologs show nuclear accumulation when oxidized in response to H_2_O_2_. Peroxiredoxins Gpx3 and Tpx1 perceive the oxidation signal and relay it to Yap1 and Pap1, respectively. To determine NapA localization we introduced a GFP tag at its C-terminus and showed that the H_2_O_2_ resistance of the corresponding strain was not affected (Figure S5), indicating the functionality of this NapA::GFP fusion. Then we analyzed NapA::GFP localization using different H_2_O_2_ concentrations. Initially, we found a very low basal signal of NapA::GFP expressed from the *napA* promoter, which increased in nuclei after 30 min of treatment with different H_2_O_2_ concentrations (not shown). Under these conditions 2 mM was the minimum concentration needed to induce NapA::GFP nuclear accumulation and therefore we used this concentration for a time-course analysis. As seen in Figure 3A, NapA::GFP starts to show nuclear localization after 20 min of treatment, which gradually increases up to 120 min. When after this time the H_2_O_2_ treatment was stop by transferring mycelia to a medium lacking H_2_O_2_, it took 6 h to observe that NapA::GFP was no longer observed in nuclei, showing instead a cytoplasm localization. This indicates that under these conditions NapA-mediated adaptation to oxidative stress is a relatively slow process. Since Δ*napA* mutants are sensitive to menadione, we also determined if menadione was able to induce NapA::GFP nuclear localization. Indeed, 10 μM menadione induced NapA nuclear accumulation after a 90 min treatment (Figure 3B).

### NapA function in the antioxidant response is independent of peroxiredoxins GpxA, TpxA, and TpxB

**Figure 4:**
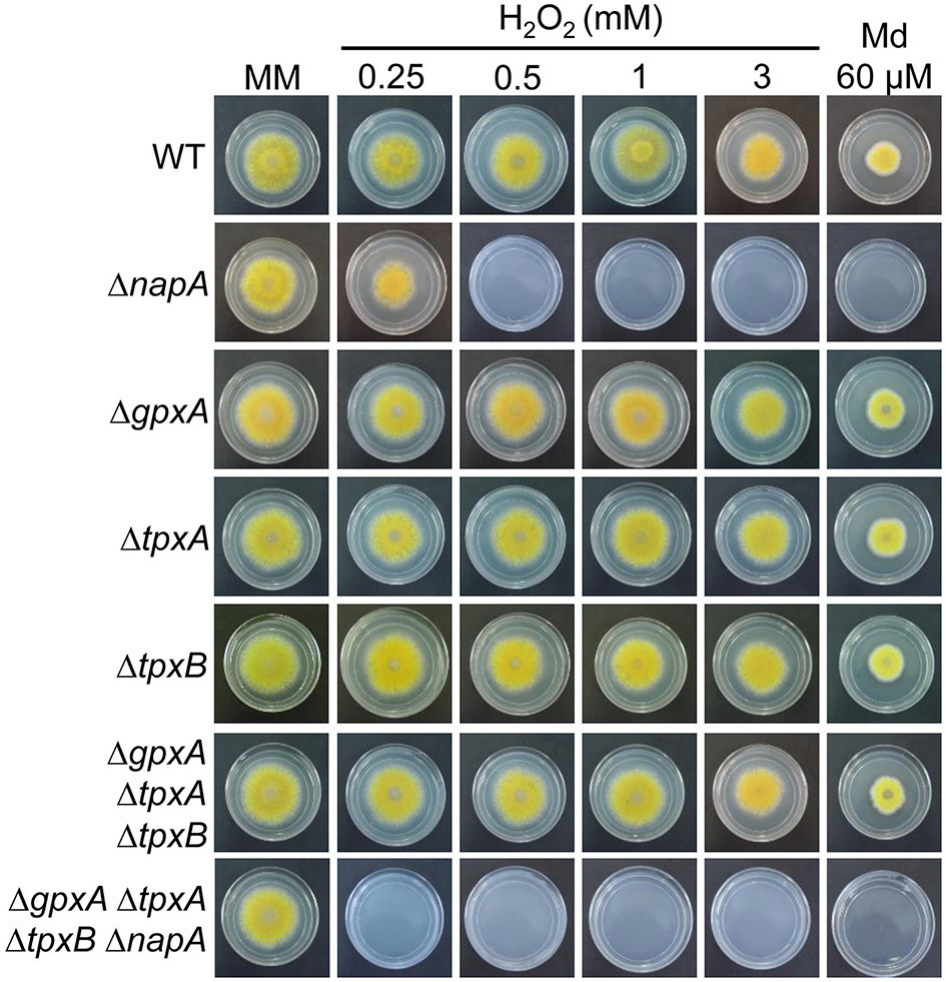
**Peroxiredoxins GpxA, TpxA and TpxB are not required for H_2_O_2_ or menadione resistance**. Conidia (1 × 10^3^) from strains CLK43 (WT), CFL7 (Δ*napA*), CAM11 (Δ*gpxA*), CAM13 (Δ*tpxA*), CAM19 (Δ*tpxB*), CAM15 (Δ*gpxA* Δ*tpxA* Δ*tpxB*), and CAM16 (Δ*gpxA* Δ*tpxA* Δ*tpxB* Δ*napA*) were inoculated on supplemented MM plates containing H_2_O_2_ or menadione (Md) at the indicated concentrations, and incubated at 37°C for 4 days.

As indicated before, *S. cerevisiae* peroxiredoxin Gpx3 and *S. pombe* Tpx1 relay the oxidation signal to Yap1 and Pap1, respectively. To determine if homologous peroxiredoxins were involved in NapA function, we searched the *A. nidulans* genome and found genes AN2846, AN10223, and AN3973 (Oh et al., 2010), which according to their closest homolog in *S. cerevisiae* are named as *gpxA* (Thön et al., 2010), *tpxA* and *tpxB*, respectively. We generated strains in which one of these peroxiredoxin genes was deleted and by sexual crosses generated triple as well as quadruple mutants containing the Δ*napA* deletion. In sharp contrast to Δ*napA* mutants, mutants lacking either a single or all 3 peroxiredoxins were not sensitive to H_2_O_2_ or menadione. However, the simultaneous inactivation of the 3 peroxiredoxins resulted in an enhancement of the sensitivity to H_2_O_2_ caused by the inactivation of NapA (Figure 4). This suggests that these 3 proteins might play a partially redundant minor function in H_2_O_2_ resistance, different from NapA.

### NapA is involved in developmental regulation

**Figure 5:**
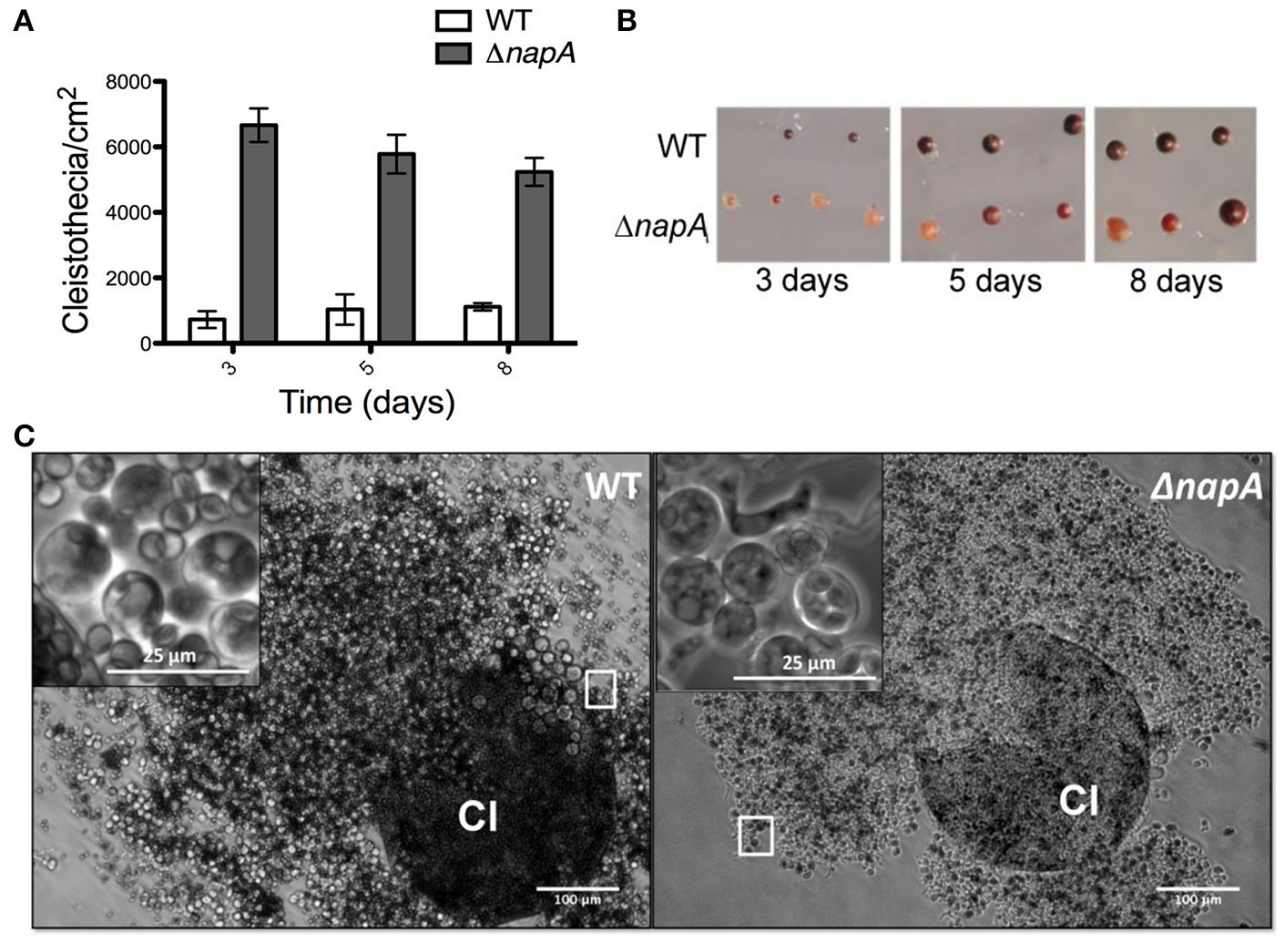
**NapA regulates sexual development. (A)** The deletion of *napA* causes premature sexual development. Conidia from strains CLK43 (WT) or CFL7 (Δ*napA*) were grown and induced to undergo sexual development as reported (Kawasaki et al., 2002). The total number of cleistothecia per fixed area was counted under a dissection microscope and used to calculate cleistothecia per cm^2^. Bars represent the standard error of the mean (SEM). **(B)** Cleistothecia from 3, 5, and 8 days from experiment in **(A)** were isolated and photographed under a dissection microscope. **(C)** Δ*napA* mutants develop unpigmented cleistothecia containing pigmented ascospores. WT and Δ*napA* cleistothecia (Cl) from 5-day old cultures were crushed and photographed under the microscope. Square areas in each picture show enlargements of asci and ascospores.

**Figure 6:**
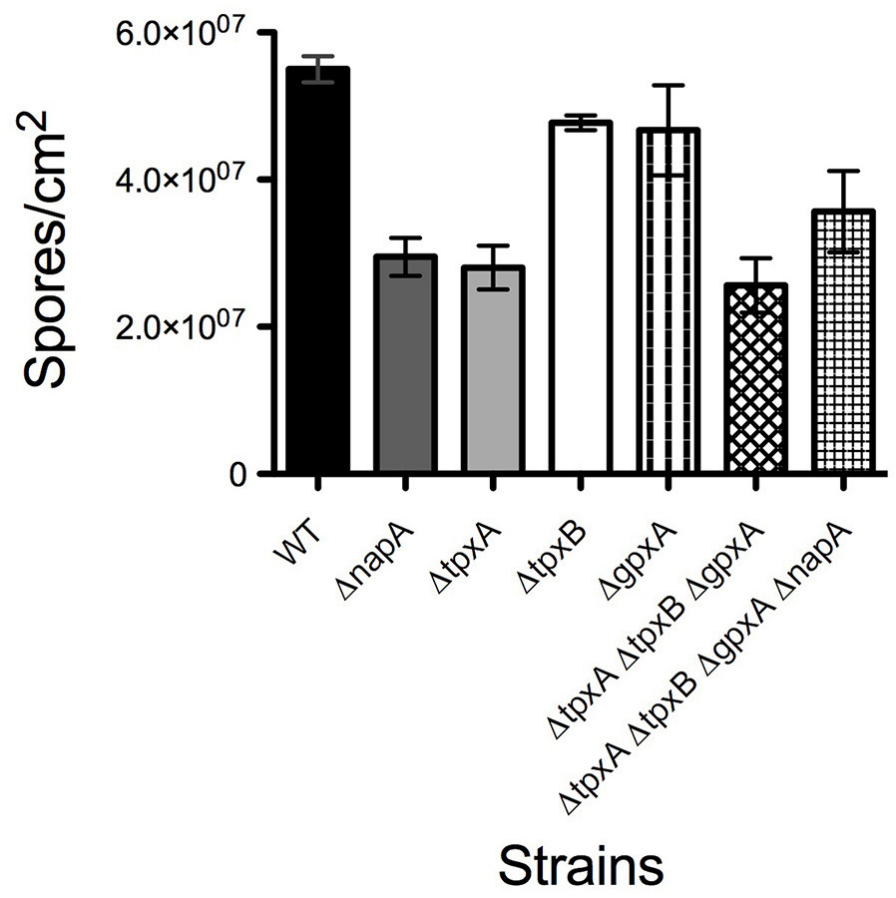
**NapA and TpxA are required for normal asexual development**. Asexual spores (1 × 10^3^) from strains CLK43 (WT), CFL7 (Δ*napA*), CAM13 (Δ*tpxA*), CAM11 (Δ*gpxA*), CAM11 (Δ*gpxB*), CAM15 (Δ*gpxA* Δ*tpxA* Δ*tpxB*), CAM16 (Δ*gpxA* Δ*tpxA* Δ*tpxB* Δ*napA*), CAM17 (Δ*alcA*), and CAM18 (Δ*alcA* Δ*napA*), were inoculated on supplemented MM and incubated at 37°C for 4 days. Total conidia were harvested and counted. Bars indicate standard deviation from three independent experiments.

Δ*napA* mutants did not show any obvious defects except that they seemed to develop more and paler cleistothecia than the WT strain. To examine this in more detail, we induced sexual development in confluent cultures and determined the number of cleistothecia as reported before (Kawasaki et al., 2002). Indeed, a Δ*napA* mutant produced about 3 times more cleistothecia than the WT strain (Figure 5A). Notably, Δ*napA* young cleistothecia were white, while older cleistothecia developed some pigmentation (Figure 5B). When observed under the microscope, it became clear that Δ*napA* cleistothecia are not pigmented (Figure 5C, right panel) but produce pigmented ascospores, which are viable (not shown). *A. nidulans veA* gene encodes a member of a fungal protein family required for cleistothecium formation (Kim et al., 2002) and regulation of secondary metabolism (Bayram et al., 2008). Since our laboratory strains contain a *veA1* allele that causes higher production of conidia and lower numbers of cleistothecia (Käfer, 1965), we also evaluated Δ*napA* sexual development phenotypes in the presence of a wild-type *veA* allele. As expected, results show higher numbers of cleistothecia in *veA*^+^ strain FGSC4 and even higher in Δ*napA veA*^+^ strains. Again, young cleistothecia were not pigmented, while older cleistothecia were non-pigmented but produced pigmented ascospores (not shown). Likewise, the presence of wild type *veA* gene did not modify Δ*napA* oxidative stress sensitivity. These results indicate that NapA represses sexual development and is needed for the expression of genes, possibly a polyketide synthetase gene, associated with the synthesis of cleistothecial melanin. When conidiation levels were determined, we found that a Δ*napA* mutant produced half of the conidia of the WT strain (Figure 6), indicating that NapA plays a role in the conidiation process. We also examined the conidiation levels of single, double and triple peroxiredoxin mutants. As shown in Figure 6, the inactivation of *gpxA* and *tpxB* had a very minor impact in conidiation. In contrast, the inactivation of *tpxA* reduced conidiation to levels similar to those observed in Δ*gpxA* Δ*tpxA* Δ*tpxB* and Δ*napA* mutants. Moreover, a quadruple Δ*gpxA* Δ*tpxA* Δ*tpxB* Δ*napA* mutant showed conidiation numbers slightly higher than those seen in single Δ*tpxA* and Δ*napA* mutants, indicating that TpxA and NapA functions in conidiation are not additive and therefore suggesting that these two proteins work in the same conidiation pathway. Notably, *tpxA* orthologs are induced by H_2_O_2_ and regulated by NapA homologs AfYap1 and Bap1 in *A. fumigatus* (Lessing et al., 2007) and *Botrytis cinerea* (Temme and Tudzynski, 2009), respectively. These results suggest that TpxA plays NapA independent and dependent roles in oxidative stress resistance and conidiation, respectively.

### NapA is required for gene regulation in conidia

To address the role of NapA in the production of fully functional conidia, we decided to carry out a transcriptomic analysis. For this, we isolated total RNA from Δ*napA* and WT intact conidia from 6-day old colonies. Two independent samples from each strain (Biological replicates) were processed for cDNA synthesis and DNA sequencing using Illumina's platform. An average of 10168 reads of 72 bp per sample representing nearly 96.3% *A. nidulans* genome lengths per sample were obtained. The two biological replicates showed a good level of correlation (r_0.966 for WT and r_0.968 for Δ*napA*) and principal component analysis showed a clear separation between Δ*napA* and WT samples. Results show changes in the expression of 284 genes with a logFC differences higher than 1.5 but lower than 2 (Table S4) and 214 genes with logFC changes higher than 2 (Tables 1, 2). The following analysis will be based mostly on these 214 genes, from which 13 genes were up regulated and 201 were down regulated in Δ*napA* conidia, as compared to WT conidia. Using gene ontology (GO) enrichment analysis, *Aspergillus* genome database AspGD (Cerqueira et al., 2014) and manual annotations, we grouped the genes regulated by NapA as shown in Tables 1, 2.

**Table 1:** **Up regulated transcripts in Δ*napA* conidia**.

**Gene**	**LogFC**	**Description**	**Domains**
**OXIDOREDUCTASES**			
AN8449	−2.1	Putative role in nitrate assimilation	Oxidoreductase molybdopterin binding domain,Mo-co oxidoreductase dimerisation domain,Cytochrome b5-like Heme/Steroid binding domain,Oxidoreductase FAD-binding domain,Oxidoreductase NAD-binding domain
AN8329	−2.0	Putative glucose oxidase-related protein	GMC oxidoreductase
**TRANSPORTER ACTIVITY**			
AN7839	−7.9	Conserved hypothetical protein	ABC transporter transmembranal, Vacuolar glutathione S-conjugate transporter
AN2475	−2.4	MFS monosaccharide transporter	SP: MFS transporter, sugar porter (SP) family,Major Facilitator Superfamily, Sugar (and other)
AN5323	−2.1	MFS sugar transporter	Major Facilitator Superfamily, Major Facilitator Superfamily, Sugar (and other)
AN7796	−2.0	Conserved hypothetical protein	Major Facilitator Superfamily
**HYDROLASES**			
AN9027	−2.7	Conserved hypothetical protein	Hydrolase activity, metallo-beta-lactamase superfamily
**UNCLASSIFIED**			
AN7834	−10.1	Conserved hypothetical protein	Heterokaryon incompatibility HET domain
AN7836	−9.4	Cysteine-rich secreted protein	Not found
AN3384	−2.4	Conserved hypothetical protein	Acetyltransferase family, possible role in trichothecene biosynthesis
AN3557	−2.0	Nucleoside metabolic process	Nucleoside phosphorylase
**HYPOTHETICAL PROTEINS WITH NO IDENTIFIED DOMAIN**			
AN2838	−2.6	Conserved hypothetical protein	Not found
AN0020	−2.3	Conserved hypothetical protein	Not found

**Table 2:** **Down regulated transcripts in Δ*napA* conidia**.

**Gene**	**LogFC**	**Description**	**Domains**
**DNA BINDING**			
AN8426	3.3	Conserved hypothetical protein	Fungal Zn(2)-Cys(6) binuclear cluster domain, Fungal specific transcription factor domain
AN3769	3.0	C6 transcription factor	Fungal specific transcription factor domain
AN7118	2.9	Putative transcription factor	Fungal specific transcription factor domain, Fungal Zn(2)-Cys(6) binuclear cluster domain, Zinc finger, C_2_H_2_ type
AN8103	2.8	Putative transcription factor	Zn(II)2Cys6 transcription factor
AN4720	2.5	conserved hypothetical protein	Zinc finger, C_2_H_2_ type
AN10897	2.4	C6 zinc finger domain-containing protein	Fungal Zn(2)-Cys(6) binuclear cluster domain
AN7061	2.3	Conserved hypothetical protein	Fungal specific transcription factor domain, Fungal Zn(2)-Cys(6) binuclear cluster domain
AN11831	2.3	Conserved hypothetical protein	Fungal Zn(2)-Cys(6) binuclear cluster domain, Fungal specific transcription factor domain
AN4626	2.3	Putative Zn(II)2Cys6 transcription factor	Has domain(s) with predicted RNA polymerase II transcription factor activity, sequence-specific DNA binding, zinc ion binding activity, role in regulation of transcription, DNA-templated and nucleus localization
AN8666	2.2	C6 finger domain-containing protein	Fungal Zn(2)-Cys(6) binuclear cluster domain
AN7346	2.0	Conserved hypothetical protein	Fungal Zn(2)-Cys(6) binuclear cluster domain, Fungal specific transcription factor domain
AN2667	2.0	C6 transcription factor	Fungal specific transcription factor domain
**OXIDOREDUCTASES**			
AN7418	5.5	FAD monooxygenase	With predicted FAD binding, oxidoreductase activity and role in metabolic process
AN7981	3.8	Ferric-chelate reductase	FAD-binding domain
AN10388	3.6	Conserved hypothetical protein	Berberine and berberine like, FAD binding domain
AN11191	3.6	Polyketide synthase	Beta-ketoacyl synthase, N-terminal domain, Beta-ketoacyl synthase, C-terminal domain, Acyl transferase domain, short chain dehydrogenase, KR domain
AN6096	3.4	Conserved hypothetical protein	Aldo/keto reductase family
AN8349	3.3	Salicylate hydroxylase	Pyridine nucleotide-disulphide oxidoreductase, FAD binding domain
AN0330	3.2	NADH-dependent flavin oxidoreductase	NADH:flavin oxidoreductase / NADH oxidase family
AN10099	3.2	Pyridoxamine phosphate oxidase	Pyridoxamine 5′-phosphate oxidase
AN2504	3.1	Conserved hypothetical protein	FAD dependent oxidoreductase
AN0618	3.1	Oxidoreductase	KR domain, short chain dehydrogenase
AN10005	3.0	Short-chain dehydrogenase/reductase SDR	Short chain dehydrogenase
AN8979	2.8	AlcA, Alcohol dehydrogenase I	Alcohol dehydrogenase GroES-like domain, Zinc-binding dehydrogenase
AN4643	2.8	Conserved hypothetical protein	Cytochrome P450
AN2704	2.8	Conserved hypothetical protein	GMC oxidoreductase
AN10296	2.8	FAD dependent oxidoreductase	FAD binding domain, flavo_cyto_c: flavocytochrome c, FAD dependent oxidoreductase, Flavin containing amine oxidoreductase
AN6414	2.8	Conserved hypothetical protein	Cytochrome P450
AN2682	2.8	12-oxophytodienoate reductase	NADH:flavin oxidoreductase / NADH oxidase family
AN1548	2.7	Short-chain dehydrogenase	Not found
AN10667	2.7	Alcohol dehydrogenase	Alcohol dehydrogenase GroES-like domain, Zinc-binding dehydrogenase
AN2723	2.7	Histidinol dehydrogenase	Histidinol dehydrogenase, hisD: histidinol dehydrogenase
AN7388	2.6	Catalase-peroxidase	Peroxidase, cat_per_HPI: catalase/peroxidase HPI
AN5287	2.6	Acyl-CoA dehydrogenase family member 11	Acyl-CoA dehydrogenase, middle domain, Acyl-CoA dehydrogenase, C-terminal domain
AN5421	2.5	Steroid monooxygenase	Not found
AN3400	2.5	Short chain type dehydrogenase	KR domain, short chain dehydrogenase
AN3206	2.5	Glucose-methanol-choline oxidoreductase	GMC oxidoreductase
AN7415	2.5	Conserved hypothetical protein	FAD binding domain
AN1034	2.5	Polyketide synthase	NAD dependent epimerase/dehydratase, short chain dehydrogenase, Methyltransferase domain, Male sterility protein, Acyl transferase domain, Phosphopantetheine attachment site, Beta-ketoacyl synthase, C-terminal domain, Beta-ketoacyl synthase, N-terminal domain
AN0027	2.5	Cyclohexanone monooxygenase	Flavin-binding monooxygenase-like, Pyridine nucleotide-disulphide oxidoreductase
AN0554	2.4	Aldehyde dehydrogenase ALDH	Aldehyde dehydrogenase family
AN5854	2.4	Oxidoreductase	2-nitropropane dioxygenase, FMN-dependent dehydrogenase, Conserved region in glutamate synthase
AN8547	2.3	GMC oxidoreductase	GMC oxidoreductase
AN2389	2.3	Ketopantoate reductase	Ketopantoate reductase PanE/ApbA C terminal, apbA_panE: 2-dehydropantoate 2-reductase, Ketopantoate reductase PanE/ApbA
AN1825	2.3	Sulfide:quinone oxidoreductase	Pyridine nucleotide-disulphide oxidoreductase
AN11096	2.3	Conserved hypothetical protein	Short chain dehydrogenase
AN5360	2.3	Conserved hypothetical protein	Cytochrome P450
AN5373	2.3	3-oxoacyl-(acyl-carrier-protein) reductase	Short chain dehydrogenase, 23BDH: acetoin reductases
AN8250	2.3	Conserved hypothetical protein	Cytochrome P450
AN4126	2.3	Aldehyde dehydrogenase	Aldehyde dehydrogenase family
AN2396	2.3	Conserved hypothetical protein	NAD dependent epimerase/dehydratase family
AN2666	2.2	Sorbitol/xylitol dehydrogenase	Zinc-binding dehydrogenase, bchC: Chlorophyll synthesis pathway, bchC, Alcohol dehydrogenase GroES-like domain
AN9315	2.2	Conserved hypothetical protein	Pyridine nucleotide-disulphide oxidoreductase
AN2335	2.2	3-hydroxyisobutyrate dehydrogenase	NAD binding domain of 6-phosphogluconate
AN4829	2.1	Alcohol dehydrogenase	Aldo/keto reductase family
AN3399	2.0	FAD binding oxidoreductase	FAD binding domain
AN5550	2.0	Conserved hypothetical protein	FAD binding domain
AN6659	2.0	Short chain dehydrogenase	KR domain, short chain dehydrogenase, NAD dependent epimerase/dehydratase
**TRANSPORTER ACTIVITY**			
AN9000	4.4	MFS transporter	Major Facilitator Superfamily
AN10891	3.8	High-affinity glucose transporter	SP: MFS transporter, sugar porter (SP) family, Sugar (and other), Major Facilitator Superfamily
AN8981	3.6	AlcS, conserved hypothetical protein	GPR1/FUN34/yaaH family
AN6019	3.5	MFS transporter	Major Facilitator Superfamily
AN12129	3.0	Conserved hypothetical protein	Major Facilitator Superfamily, Sugar (and other) transporter
AN2368	3.0	Membrane transporter	Major Facilitator Superfamily
AN9010	2.8	MFS nicotinic acid transporter Tna1	Major Facilitator Superfamily
AN2699	2.8	Conserved hypothetical protein	Major Facilitator Superfamily, Ion channel regulatory protein UNC-93
AN7380	2.7	Conserved hypothetical protein	Major Facilitator Superfamily
AN11211	2.6	Uracil permease	Permease for cytosine/purines, uracil, thiamine, allantoin, ncs1: NCS1 nucleoside transporter family
AN5275	2.6	Choline transporter	Amino acid permease
AN2665	2.5	MFS sugar transporter	Major Facilitator Superfamily, Major Facilitator Superfamily, Sugar (and other)
AN2358	2.5	Conserved hypothetical protein	Sugar (and other), Major Facilitator Superfamily
AN8995	2.5	Conserved hypothetical protein	Major Facilitator Superfamily
AN3207	2.5	Amino acid transporter	Transmembrane amino acid transporter protein
AN9165	2.4	Conserved hypothetical protein	Sugar (and other) transporter, Major Facilitator Superfamily
AN3352	2.4	Conserved hypothetical protein	ncs1: NCS1 nucleoside transporter family, Permease for cytosine/purines, uracil, thiamine, allantoin
AN2201	2.4	Proline permease	Amino acid permease
AN8352	2.4	Carbixilic Transporter	Not found
AN8941	2.4	Na/K ATPase alpha 1 isoform	Cation transporting ATPase, C-terminus, haloacid dehalogenase-like hydrolase, ATPase_P-type: HAD ATPase, P-type, family IC, E1-E2 ATPase
AN2959	2.3	Allantoate transporter	Major Facilitator Superfamily
AN6063	2.2	MFS transporter	Major Facilitator Superfamily, 2_A_01_02: Multidrug resistance protein, Sugar (and other) transporter, pump (TRI12)
AN12222	2.2	Conserved hypothetical protein	Major Facilitator Superfamily
AN8955	2.2	Conserved hypothetical protein	Major Facilitator Superfamily
AN3503	2.2	Allantoate permease	Major Facilitator Superfamily
AN5187	2.1	Na(+)/H(+) antiporter	Sodium/hydrogen exchanger family
AN2814	2.1	MFS lactose permease	Major Facilitator Superfamily, SP: MFS transporter, sugar porter (SP) family
AN6960	2.1	Conserved hypothetical protein	WD domain, G-beta repeat, Ankyrin repeat, CorA-like Mg2+ transporter
AN6451	2.1	Conserved hypothetical protein	Major Facilitator Superfamily, Sugar (and other) transporter, 2_A_01_02: Multidrug resistance protein, Fungal trichothecene efflux pump (TRI12)
AN7067	2.0	Conserved hypothetical protein	Major Facilitator Superfamily, SP: MFS transporter, sugar porter (SP) family, Sugar (and other)
AN10305	2.0	Vacuolar membrane ATPase C	V_ATP_synt_C: V-type ATPase, C subunit, ATP synthase subunit C
AN9336	2.0	Conserved hypothetical protein	Major Facilitator Superfamily
**HYDROLASES**			
AN2690	4.0	Conserved hypothetical protein	Glycosyl hydrolases family 16
AN10346	3.8	Cutinase	Cutinase, Phospholipase/Carboxylesterase
AN3402	3.8	Alpha-amylase	Starch binding domain, Domain of unknown function (DUF1966), Alpha amylase, catalytic domain
AN8899	3.8	1-aminocyclopropane-1-carboxylate deaminase	Pyridoxal-phosphate dependent enzyme, ACC_deam: 1-aminocyclopropane-1-
AN5422	3.8	Beta-lactamase	Beta-lactamase
AN6097	3.7	Conserved hypothetical protein	3-carboxy-cis,cis-muconate lactonizing enzyme
AN2834	3.5	Conserved hypothetical protein	GDSL-like Lipase/Acylhydrolase
AN1792	3.5	GDSL Lipase/Acylhydrolase	GDSL-like Lipase/Acylhydrolase
AN2539	3.3	Haloalkanoic acid dehalogenase	HAD_type_II: haloacid dehalogenase, type II, HAD-SF-IA-v2: HAD hydrolase, family IA, variant 2
AN3777	3.1	Endo-beta-1,6-glucanase	Cellulase (glycosyl hydrolase family 5)
AN0022	3.1	Alpha-galactosidase/alpha-n-acetylgalactosaminidase	Melibiase
AN11051	3.0	Extracellular chitosanase CsnC	Fungal chitosanase
AN3613	2.8	Xylanase	Glycosyl hydrolases family 11
AN5060	2.7	Conserved hypothetical protein	Has domain(s) with predicted arylformamidase activity and role in tryptophan catabolic process to kynurenine
AN11219	2.7	Glutamyl-tRNA(Gln) amidotransferase subunit A	Amidase
AN10333	2.7	Metallopeptidase, putative	Has domain(s) with predicted hydrolase activity and role in metabolic process
AN6656	2.6	Endo-polygalacturonase D	Glycosyl hydrolases family 28
AN9518	2.5	Haloalkanoic acid dehalogenase	HAD_type_II: haloacid dehalogenase, type II, HAD-SF-IA-v2: HAD hydrolase, family IA, variant 2
AN7275	2.5	Xylosidase/glycosyl hydrolase	Glycosyl hydrolases family 43
AN0181	2.4	Conserved hypothetical protein	Thioesterase superfamily
AN8977	2.4	AlcP, Conserved hypothetical protein	Strictosidine synthase, SMP-30/Gluconolactonase/LRE-like region
AN5608	2.4	Conserved hypothetical protein	Carboxylesterase
AN1043	2.3	Conserved hypothetical protein	Glycosyl hydrolases family 43
AN2779	2.2	Dipeptidase	Amidohydrolase family
AN2388	2.2	Beta-1,4-endoglucanase	Not found
AN1826	2.1	Metallo-beta-lactamase domain-containing protein	Metallo-beta-lactamase superfamily
AN2424	2.1	Beta-N-acetylhexosaminidase	Glycosyl hydrolase family 20, catalytic domain
AN7911	2.1	Conserved hypothetical protein	Amidohydrolase
AN2777	2.0	Fumarylacetoacetate hydrolase	Fumarylacetoacetate (FAA) hydrolase family, alpha/beta hydrolase fold
**UNCLASSIFIED**			
AN2587	8.7	Conserved hypothetical protein	CFEM domain
AN11810	3.7	Putative GNAT-type acetyltransferase	Not found
AN8980	3.4	AlcM, conserved hypothetical protein	Not found
AN8092	3.3	Conserved hypothetical protein	pfs domain Potential Cdc28p substrate
AN0180	3.0	Putative Enoyl-CoA hydratase	Enoyl-CoA hydratase/isomerase family
AN7539	3.0	Hydrophobin	Fungal hydrophobin
AN8913	3.0	Predicted glycosylphosphatidylinositol (GPI)-anchored protein	Not found
AN8614	2.7	Conserved hypothetical protein	Cupin domain
AN9354	2.7	Putative transcription factor	NmrA-like family Transcription factor
AN2954	2.6	Extracellular serine-rich protein	Not found
AN1982	2.5	Cell cycle regulatory protein, putative	WD domain
AN5273	2.5	Hydroxymethylglutaryl-CoA lyase	Enoyl-CoA hydratase/isomerase family,HMGL-like
AN8468	2.2	Wd-repeat protein	NACHT domain, WD domain
AN9297	2.2	FAD binding, oleate hydratase activity and role in fatty acid metabolic process	Not found
AN8622	2.2	Phosphodiesterase/alkaline phosphatase D	PhoD-like phosphatase
AN1046	2.2	Chitin synthase D	Chitin synthase
AN2022	2.2	Putative heterokaryon incompatibility protein	Not found
AN11080	2.1	DMATS type aromatic prenyltransferase	Tryptophan dimethylallyltransferase, arom_pren_DMATS: aromatic prenyltransferase, DMATS type
AN3730	2.1	1,3-beta-glucanosyltransferase	X8 domain, Glycolipid anchored surface protein (GAS1)
AN0017	2.1	Conserved hypothetical protein	CutC family
AN2623	2.1	Acyl-coenzyme A:6-aminopenicillanic-acid-acyltransferase	Not found
AN5833	2.1	Acyl-CoA synthetase	AMP-binding enzyme, Domain of unknown function (DUF3448)
AN7279	2.1	GPI anchored protein poly(beta-D-mannuronate) lyase activity	Not found
AN2346	2.1	Conserved hypothetical protein	Pfs domain
AN10302	2.1	Hypothetical protein	NACHT domain
AN3159	2.0	Conserved hypothetical protein	RasGEF domain
AN8368	2.0	Conserved hypothetical protein	Spherulation-specific family 4
AN2778	2.0	Conserved hypothetical protein	Cytochrome b5-like Heme/Steroid binding domain
**HYPOTHETICAL PROTEINS WITH NO IDENTIFIED DOMAINS**			
AN2676	4.9	Conserved hypothetical protein	Not found
AN7203	3.5	Conserved hypothetical protein	Not found
AN8369	3.4	Predicted protein	Not found
AN11341	3.2	Predicted protein	Not found
AN10086	3.1	DUF1446 domain-containing protein	Protein of unknown function (DUF1446)
AN9331	3.1	Conserved hypothetical protein	Not found
AN6797	3.1	Conserved hypothetical protein	Not found
AN4122	3.1	Conserved hypothetical protein	Protein of unknown function
AN2322	3.0	Conserved hypothetical protein	Not found
AN10360	2.9	Conserved hypothetical protein	Not found
AN9323	2.9	Conserved hypothetical protein	Not found
AN7261	2.8	Conserved hypothetical protein	Not found
AN5190	2.8	Conserved hypothetical protein	Not found
AN2700	2.8	Conserved hypothetical protein	Not found
AN0972	2.7	Conserved hypothetical protein	Not found
AN2750	2.7	Conserved hypothetical protein	Not found
AN1928	2.7	Conserved hypothetical protein	Not found
AN12341	2.7	Hypothetical protein	Not found
AN1042	2.7	Conserved hypothetical protein	Not found
AN10326	2.6	Predicted protein	Not found
AN7333	2.6	Conserved hypothetical protein	Not found
AN12061	2.6	Conserved hypothetical protein	Not found
AN8240	2.6	Conserved hypothetical protein	Not found
AN7985	2.5	Conserved hypothetical protein	Not found
AN11290	2.5	Conserved hypothetical protein	Not found
AN7270	2.5	Conserved hypothetical protein	Not found
AN4554	2.5	Conserved hypothetical protein	Not found
AN11809	2.5	Conserved hypothetical protein	Not found
AN2041	2.4	Conserved hypothetical protein	Not found
AN10048	2.4	Conserved hypothetical protein	Not found
AN1925	2.4	Conserved hypothetical protein	Not found
AN8994	2.4	Conserved hypothetical protein	Not found
AN11281	2.4	Conserved hypothetical protein	Not found
AN12238	2.3	Conserved hypothetical protein	Not found
AN10320	2.3	Conserved hypothetical protein	Not found
AN9521	2.2	Conserved hypothetical protein	Not found
AN7124	2.2	Conserved hypothetical protein	Not found
AN4319	2.2	Conserved hypothetical protein	Not found
AN0005	2.2	Hypothetical protein	Not found
AN4136	2.2	Conserved hypothetical protein	Not found
AN11585	2.2	Conserved hypothetical protein	Not found
AN7976	2.1	Conserved hypothetical protein	Not found
AN11872	2.1	Predicted protein	Not found
AN8148	2.1	Conserved hypothetical protein	Not found
AN6648	2.1	Conserved hypothetical protein	Not found
AN11777	2.1	Predicted protein	Not found
AN11865	2.1	Conserved hypothetical protein	Not found
AN0481	2.1	Conserved hypothetical protein	Not found
AN8008	2.1	Conserved hypothetical protein	Not found
AN1649	2.1	Conserved hypothetical protein	Not found
AN7099	2.1	Conserved hypothetical protein	Not found
AN8740	2.1	Conserved hypothetical protein	Not found
AN4642	2.0	Conserved hypothetical protein	Not found
AN0539	2.0	Conserved hypothetical protein	Not found

**Figure 7:**
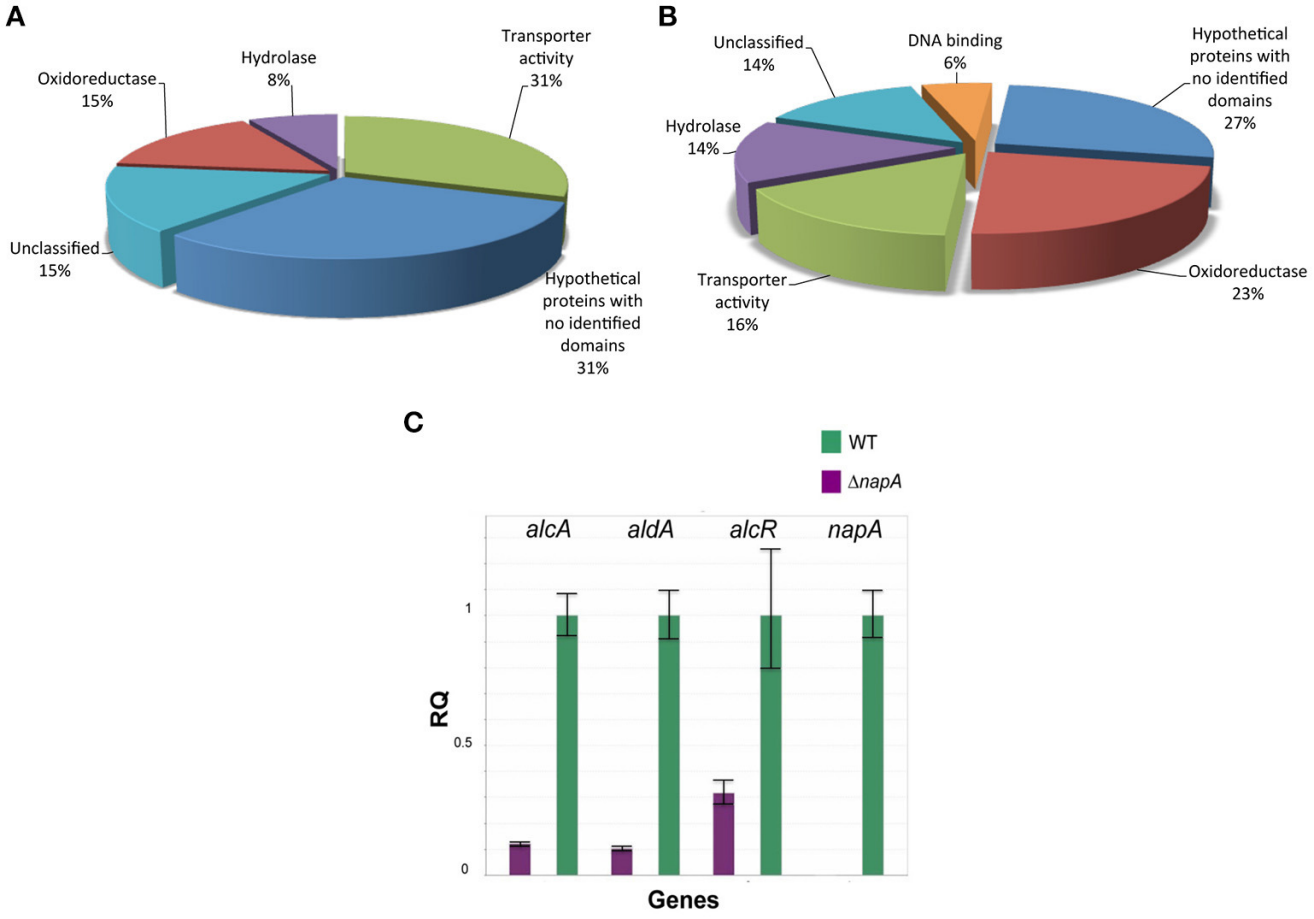
**NapA is required for gene regulation in asexual spores**. Transcriptomic analysis reveals that NapA is needed for the negative regulation of at least 13 genes **(A)** and the positive regulation of at least 201 genes **(B)** in conidia. **(C)** Confirmation of NapA-dependent expression of ethanol utilization genes during conidiation by qPCR. Genes *alcA, aldA* and *alcR* were used to confirm transcriptome results from conidia. Gene expression was normalized to H2B histone transcript levels. Data are mean values of three independent biological replicates. Bars represent the standard deviation (SD).

Our results show that the number of genes negatively regulated by NapA is small (13), compared with the number of genes positively regulated by NapA (201). Among genes most highly repressed by NapA, there is the putative HET (heterokaryon incompatibility) containing domain protein AN7834, the cysteine-rich secreted protein AN7836 and the putative ABC transporter AN7839, which seems to represent the only NapA-regulated ABC-type transporter. In addition, this group includes genes for 3 additional putative transporters (MSF type), 2 oxidoreductases, 1 hydrolase, 1 acetyltransferase, 1 protein with a nucleoside phosphorylase domain and 2 proteins with no identifiable domains (Figure 7A, Table 1).

Among the 201 genes positively regulated by NapA (Figure 7B, Table 2), 12 encode putative (TFs) not yet characterized in *A. nidulans*, most of them belonging to the Zn(2)-Cys(6) DNA-binding domain family. Remarkably, 46 genes encode different types of oxidoreductases, including multiple monooxygenases, dehydrogenases, several members of the P450 cytochrome drug detoxifying enzyme family and the catalase peroxidase CatD/CpeA (AN7388). Other genes encode proteins with lipase, peptidase and other hydrolytic activities. 32 genes encode putative membrane proteins involved in the transport of sugars, drugs, amino acids, metals or other metabolites, 21 of which are members of the major facilitator superfamily (MSF). 29 genes encode proteins with predicted or confirmed hydrolytic enzyme activity. Notably, several of these are related to the hydrolysis of plant cell-wall complex carbohydrates, such as putative cutinase AN10346, alpha-amylase AN3402, endo-beta-1,6-glucanase AN3777, xylanase AN3613, endo-polygalacturonase AN6656, xylosidase AN7275, beta-1,4-endoglucanase AN2388, while xylose inducible alpha-galactosidase *aglC*/AN8138, alpha-xylosidase *agdD*/AN7505, putative beta-glucosidase *bglH/*AN3903, putative alpha-amylase *amyC*/AN4507 were detected as NapA regulated with logFC values between 2 and 1.5 (Table 2 and Table S4). Other hydrolases like putative beta-lactamases AN5422 and AN1826 might be related to antibiotic degradation.

Twenty eight genes encode proteins that have recognizable domains but did not belong to the previous categories. Among these, AN2587 shows the highest LogFC value (8.7), and encodes a putative membrane protein with a cysteine-rich CFEM domain, recently shown to be involved in Fe^3+^ heme acquisition (Nasser et al., 2016). This and the fact NapA also regulates ferric reductase AN7981, putative heme binding protein AN2778 and NRPS SidC (AN0607, siderophore biosynthesis; Table S4) genes supports a NapA role in iron acquisition. Also notable is NapA regulation of several genes involved in the biosynthesis of secondary metabolites (Tables 2 and Table S4), such as sterigmatocystin (*stcU*/AN7806), penicillin (*ipnA*/AN2622), monodictyphenone and prenyl xanthones (Bok et al., 2009; Sanchez et al., 2011; Andersen et al., 2013) (*xptB*/AN12402, *mdpB*/AN10049, *mdpC*/AN0146 and *mdpD*/AN0147), all with clear antimicrobial activity. Other genes are involved in the biosynthesis of asperfuranone (*afoE/*AN1034) or unknown metabolites (PKS AN11191). In contrast, NapA has been reported to repress the production of secondary metabolites in *A. parasiticus* (Reverberi et al., 2008) and *A. nidulans* (Yin et al., 2013) during growing conditions, suggesting that NapA regulates secondary metabolism in opposite ways during growth and conidiation. Finally, NapA regulates at least 54 genes encoding hypothetical proteins with no recognizable domains (Figure 7B, Table 2).

In summary, our transcriptomic results support a model in which during conidiation NapA regulates functions associated with the successful germination of conidia in natural environments. In the case of *A. nidulans* saprophytic life style this would include a large group of enzymes to degrade plant complex carbohydrates, proteins to transport the corresponding derived sugars and enzymes needed to transform them into acetyl-CoA. NapA also regulates the production of secondary metabolites, such as penicillin, which would prevent growth from competing organisms, as well as enzymes and transporters to detoxify drugs produced by competitors. In the same line, NapA regulates enzymes and proteins involved in iron scavenging, such as those involved in siderophore biosynthesis, iron reduction and transport, and heme acquisition. As such functions are also important during pathogenic interactions, we propose that these and not NapA ROS-detoxification roles, might be relevant for NapA critical virulence roles (Molina and Kahmann, 2007; Guo et al., 2011; Huang et al., 2011), specially when conidia mediate the infection process.

### NapA is required for *alc* gene expression and the utilization of ethanol, arabinose, and fructose as sole carbon sources

It called our attention that the mRNA levels of genes *alcR* (AN8978; Table S4), *alcA* (AN8979), *alcU* (AN8982; Table S4) and *aldA* (AN0554), all members of the well-studied ethanol system (Fillinger and Felenbok, 1996), were clearly reduced in Δ*napA* conidia. The ethanol system is composed by genes *alcP, alcR, alcO, alcA, alcM, alcS*, and *alcU*, all clustered in chromosome VII and *aldA* located in chromosome VIII. *alc* genes share the same regulation, being strongly induced by the physiological inducer acetaldehyde and the non-physiological inducer 2-butanone (Flipphi et al., 2002). They are also subject to strict control by the transcriptional activator AlcR and are repressed by glucose via the CreA repressor (Fillinger and Felenbok, 1996). However, only AlcR, alcohol dehydrogenase I AlcA, and aldehyde dehydrogenase AldA are required for ethanol utilization, and the specific function of the other *alc* genes is unknown. Yet, AlcS is a membrane protein with homology to acetate transporters that is nevertheless dispensable for growth on ethanol, acetaldehyde or acetate (Flipphi et al., 2006). Therefore, we decided to confirm *alcR, alcA* and *aldA* expression results by using qPCR. Results in Figure 7C show that indeed, the accumulation of the corresponding mRNAs in conidia depends on NapA.

**Figure 8:**
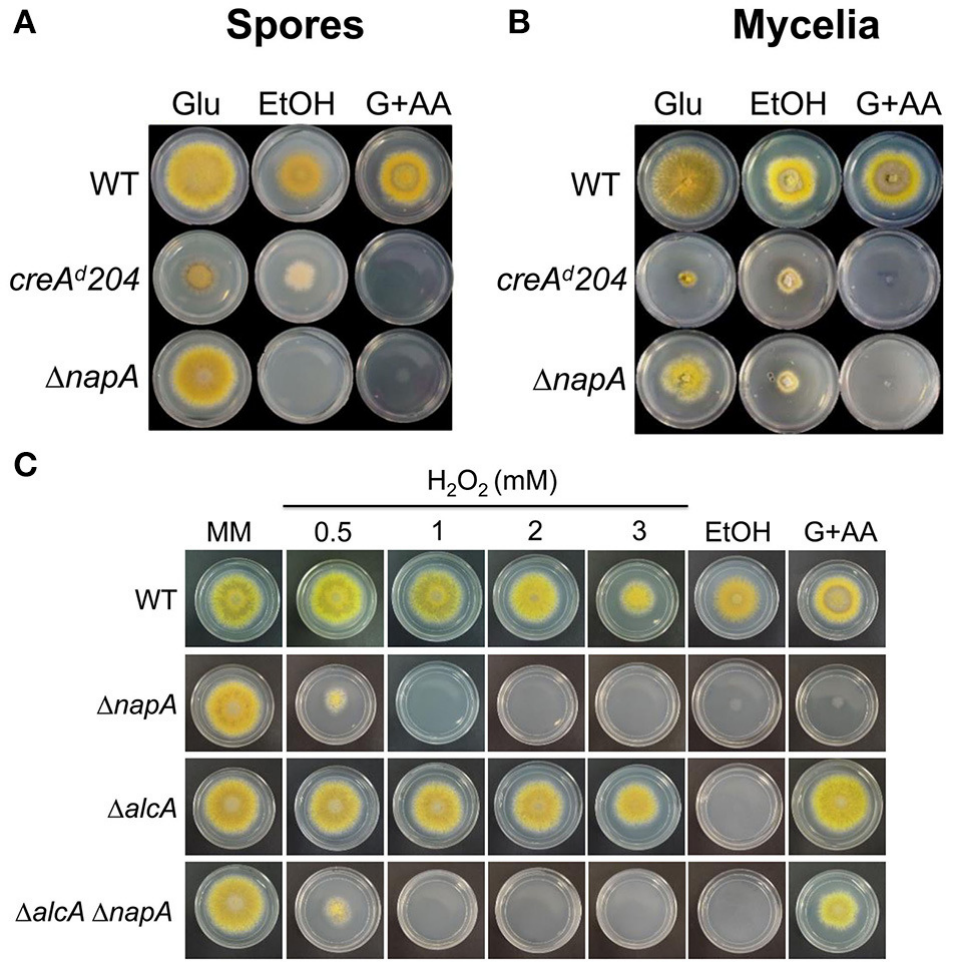
**NapA is required for the ethanol utilization and for resistance to Allyl alcohol. (A)** Conidia (1 × 10^3^) from strains CLK43 (WT), CFL7 (Δ*napA*) and MH440 (*creA*^*d*^*204*) were inoculated on supplemented MM plates containing either 1% glucose, 1% ethanol or 1% glucose plus 5 mM Allyl alcohol (AA) and were incubated at 37°C for 4 days. **(B)** Mycelial plugs cut from the growing edge of 5-day colonies from the strains in **(A)** were transferred to the indicated media and incubated at 37°C for 4 days. **(C)** Conidia (1 × 10^3^) from strains CLK43 (WT), CFL7 (Δ*napA*), CAM17 (Δ*alcA*), and CAM18 (Δ*alcA* Δ*napA*) were inoculated on supplemented MM plates containing either H_2_O_2_ at the indicated concentrations or 1% glucose (MM), 1% ethanol or 1% glucose plus 5 mM allylic alcohol (AA) and incubated at 37°C for 4 days.

To test the physiological significance of NapA-mediated *alc* gene regulation, we examined the ability of Δ*napA* mutants to grow on ethanol as sole carbon source. As control we included a *creA*^*d*^*204* mutant, which shows derepressed expression of *alcA* in the presence of glucose (Shroff et al., 1996). Since AlcA transforms allyl alcohol (AA) into the toxic compound acrolein, this *creA* mutant is unable to grow on glucose plus AA (Figures 8A,B). Notably, Δ*napA* conidia and mycelia were unable to grow on ethanol, supporting NapA function in proper *alcA* expression. However, the Δ*napA* mutant was also unable to grow on glucose plus AA (Figures 8A,B). AA/acrolein causes oxidative stress and induces Yap1 activation in *S. cerevisiae* (Kwolek-Mirek et al., 2009; Golla et al., 2015) and therefore it might result particularly toxic for the Δ*napA* mutant. To test this, we generated Δ*alcA* and Δ*napA* Δ*alcA* mutants and examined them for ethanol utilization and AA resistance. As seen in Figure 8C, the Δ*alcA* mutant is not sensitive to H_2_O_2_, is unable to grow on ethanol and is fully resistant to AA. In contrast, a Δ*napA* Δ*alcA* mutant is sensitive to H_2_O_2_, unable to grow on ethanol and more sensitive to AA than the Δ*alcA* mutant, supporting the idea that Δ*napA* mutants are *per se* more sensitive to AA. The failure to grow in 1% ethanol was not remediated by the presence of the antioxidant N-acetylcysteine (5 mM), suggesting that such failure is related to the reduced expression of the *alc* genes. However, it cannot be excluded that this might be also related to an increased ethanol sensitivity of the Δ*napA* mutants. In summary, these results show that NapA is required for expression of the *alc* regulon during asexual development and for growth on ethanol, adding a new level of regulation to this well know pathway.

**Figure 9:**
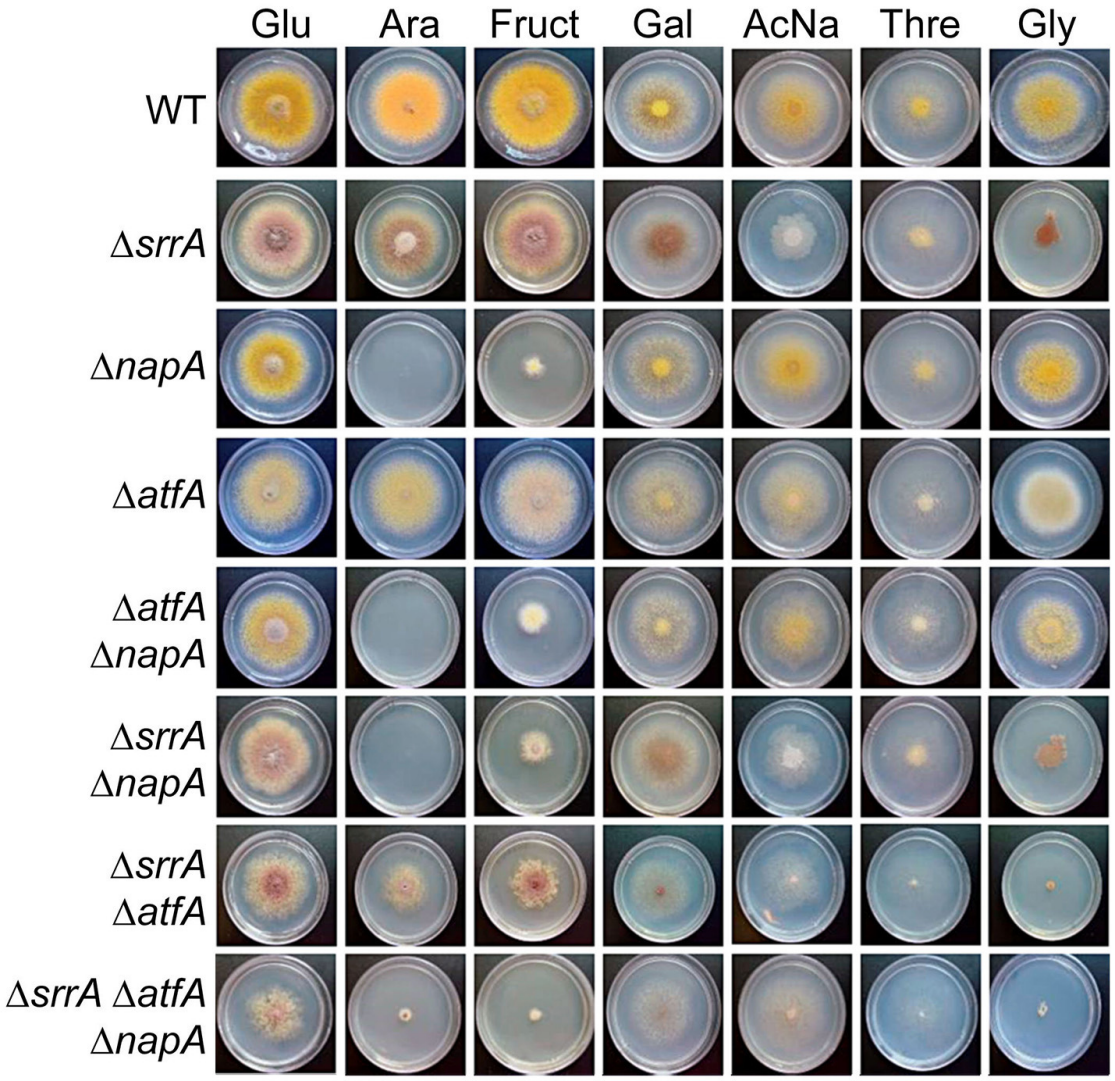
**NapA is also necessary for the utilization of arabinose and fructose as sole carbon sources**. Conidia (1 × 10^3^) from strains CLK43 (WT), COSsrrA3 (Δ*srrA*), CFL7 (Δ*napA*), TFLΔatfA-04 (Δ*atfA*), CAM7 (Δ*atfA* Δ*napA*), CAM6 (Δ*srrA* Δ*napA*), CAM8 (Δ*srrA* Δ*atfA)*, and CAM9 (Δ*srrA* Δ*atfA* Δ*napA*) were inoculated on supplemented MM plates containing either glucose (Glu), arabinose (Ara), fructose (Fruct), galactose (Gal), sodium acetate (AcNa), threonine (Thre) or 1% glycerol (Gly) as sole carbon sources and incubated at 37°C during 4 days.

These results prompted us to determine if NapA and TFs SrrA and AtfA were also required for conidia germination and growth on ethanol or other alternative carbon sources. As seen in Figure 9, NapA was also required for arabinose and fructose utilization, while SrrA was partially required for glycerol utilization and AtfA was dispensable for the utilization of all carbon sources tested. These results indicate that NapA plays an important role in the utilization of carbon sources alternative to glucose.

### NapA is localized in nuclei during growth in ethanol or glucose starvation

**Figure 10:**
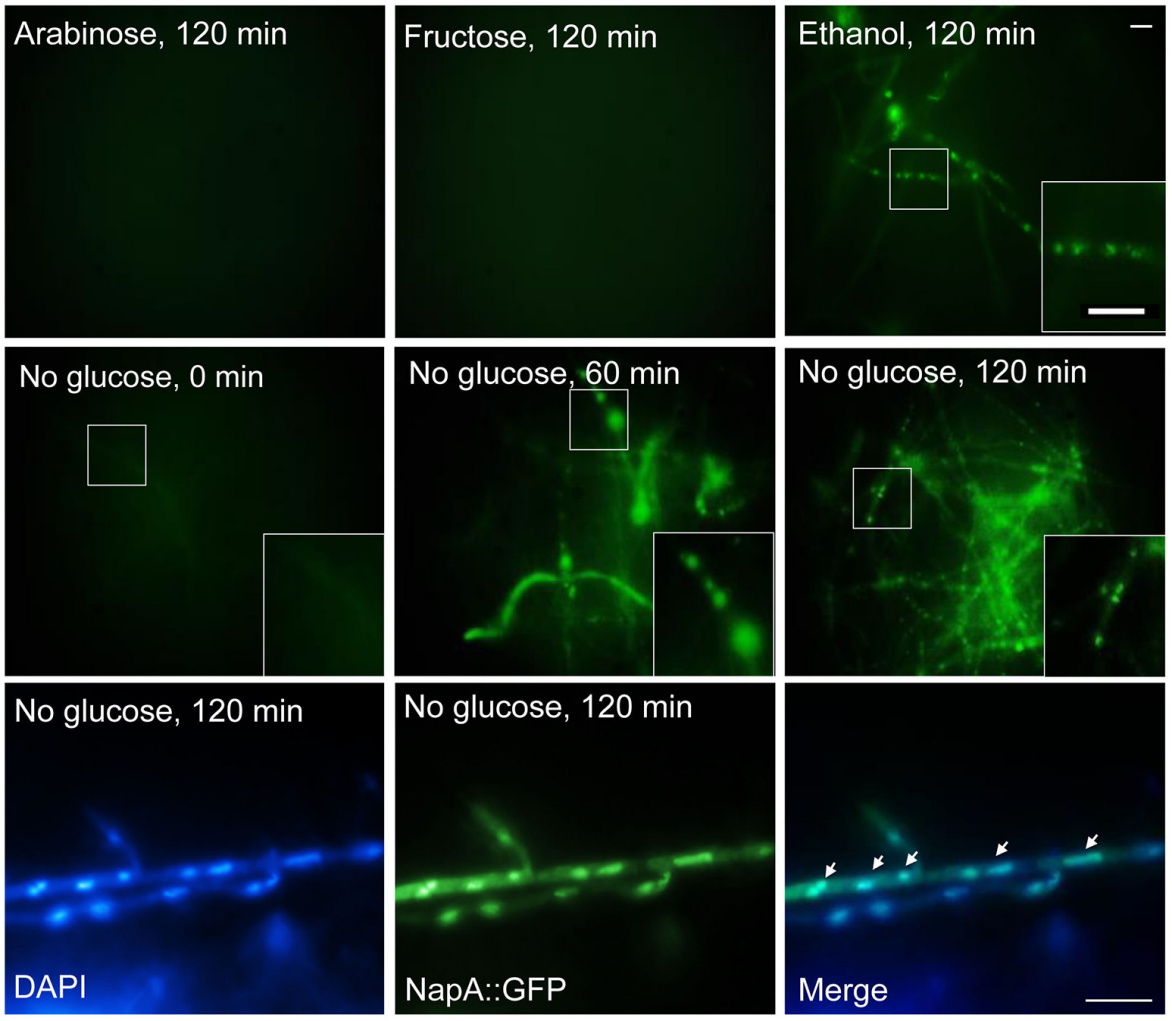
**Glucose starvation induces NapA nuclear localization**. NapA does not accumulate in nuclei during growth in arabinose or fructose as sole carbon sources but it does accumulate in nuclei during glucose starvation. Conidia from strain CAM20 (NapA::GFP) were grown for 18 h in MM at 37°C and then mycelia was shifted to MM with or without glucose or with fructose or glycerol as carbon source for indicated times (0–120 min). Mycelial samples were observed *in vivo* and photographed every 60 min using Epifluorescence microscopy. Lower panel shows nuclei (DAPI) and NapA::GFP fluorescence in mycelia starved for glucose during 120 min, fixed and photographed. Larger square areas in each picture show enlargements of the areas indicated by smaller squares.

Having found that NapA is necessary for proper growth in arabinose and fructose, we explored NapA localization during growth in poor carbon sources, as well as during glucose starvation. Results in Figure 10 show that as it occurs during growth in glucose, NapA expression is very low during growth in arabinose and fructose as sole carbon sources. In contrast, NapA::GFP expression was induced during growth in ethanol, as well as during glucose starvation, and NapA::GFP nuclear localization was detected between 60 and 120 min after shifting to ethanol or glucose lacking media. In addition, we found that a osmotic stress treatment with 1 M NaCl is able to induce NapA nuclear localization under normal glucose growth conditions (Figure S7), despite the fact that Δ*napA* mutants are not sensitive to osmotic stress.

## Discussion

### AtfA, SrrA, and NapA play non-redundant and differential roles in the antioxidant response

At least three pathways, mediated by TFs, AtfA, SrrA and NapA, are involved in fungal responses to oxidative and other types of stress and we have studied them here in the same organism. We have reported that four catalases present in *A. nidulans* display differential regulation during growth, stress and development (Navarro et al., 1996; Navarro and Aguirre, 1998; Kawasaki and Aguirre, 2001). Notably, catalase CatA is found only in asexual and sexual spores, despite the fact that *catA* mRNA accumulates under many stress conditions (Navarro and Aguirre, 1998; Lara-Rojas et al., 2011). Our results here show that TFs, AtfA, SrrA and NapA are essential components in such catalase gene regulation. NapA and SrrA are both required for CatB induction by H_2_O_2_, only AtfA is required for CatA expression in conidia and none of them is required for CatB induction during late stationary phase of growth, while CatD/CpeA activity levels are influenced by SrrA. The phenotypic analysis of oxidative stress resistance using Δ*napA*, Δ*srrA*, and Δ*atfA* single, double and triple mutants also showed that TFs NapA, SrrA, and AtfA play differential roles in oxidative stress resistance.

The functions of Ap1-like TFs in different fungi show both similarities and differences. For example and in contrast to our results, *yapA* deletion in symbiotic fungus *Epichloë festucae* causes sensitivity to H_2_O_2_ and *t*-BOOH in conidia but not in mycelia and YapA, instead of AtfA, is required for expression of the spore-specific catalase CatA (Cartwright and Scott, 2013). *Neurospora crassa* mutants lacking the NapA homolog *NcAp-1* were reported as showing no sensitivity to osmotic stress and only a slight sensitivity to H_2_O_2_ (Takahashi et al., 2010), although recent work showed that mutants were sensitive to osmotic stress, cadmium and H_2_O_2_ (Tian et al., 2011). In *Magnaporthe oryzae* inactivation of NapA homolog MoAP1 causes only a mild sensitivity to H_2_O_2_ (Guo et al., 2011), while *Ustilago maydis yap1* null mutants are sensitive to H_2_O_2_ and their virulence is significantly reduced (Molina and Kahmann, 2007). *A. fumigatus, yap1* null mutants are also sensitive to H_2_O_2_ and menadione but not affected in pathogenicity (Lessing et al., 2007).

Like in the case of Yap1 and Pap1, the activation of NapA homologs by H_2_O_2_ results in nuclear accumulation in all filamentous fungi where this has been studied, and in *U. maydis* two Yap1 conserved cysteines (Cys-399 and Cys-407) were shown to be crucial for both, nuclear accumulation and functionality (Molina and Kahmann, 2007). An additional level of redox regulation has been proposed in *A. nidulans*, where the CCAAT-binding complex (CBC) represses *napA* expression under low ROS levels, while high ROS levels result in oxidation of two cysteines in HapC CBC subunit, and the transcriptional activation of *napA* (Thön et al., 2010). We found that in addition to H_2_O_2_ and menadione, osmotic stress (Figure S7), glucose starvation stress and growth on ethanol also induced NapA nuclear localization, supporting the idea that different types of stress can all lead to oxidative stress (Hansberg and Aguirre, 1990).

### GpxA, TpxA, and TpxB peroxiredoxin function in the antioxidant response and conidiation

We analyzed the role of Gpx3 peroxiredoxin homolog GpxA and Tpx1 homologs TpxA and TpxB in NapA antioxidant and developmental functions. We found that none of these peroxiredoxins was required for H_2_O_2_ or menadione resistance and therefore are unnecessary for NapA activation. This is consistent with results in *E. festucae*, where peroxiredoxins Gpx3 (GpxA) and Tpx1 (TpxA) were not needed for YapA H_2_O_2_-induced nuclear accumulation (Cartwright and Scott, 2013). On the contrary, *M. oryzae* mutants lacking Gpx3 functional homolog MoHYR1 are sensitive to H_2_O_2_, fail to express several genes related to the antioxidant response and show reduced virulence. However, initial data showing that *MoYap1* mutants are no affected in pathogenicity indicates that MoHYR1 functions in virulence are not mediated by MoYap1 (Huang et al., 2011). In *A. fumigatus* AfYap1 also accumulates in the nuclei in response to H_2_O_2_ and notably, GpxA homolog AspF3 and TpxA require AfYap1 for its H_2_O_2_-mediated induction (Lessing et al., 2007). These and our results suggest that NapA might be required for H_2_O_2_-induced expression of *gpxA, tpxA* and *tpxB*. A minor direct or indirect role for these peroxiredoxins in H_2_O_2_ detoxification is indicated by the fact that in a Δ*napA* background, the simultaneous inactivation of Gpx3, TpxA, and TpxB resulted in increased sensitivity to H_2_O_2_. Our results suggest that TpxA and NapA regulate conidiation through the same pathway. One possibility is that NapA mediates TpxA induction during conidiation, although we did not detect *tpxA* mRNA in our transcriptomic experiments, and TpxA could in turn regulate other activities needed for full sporulation.

### NapA regulates development

We reported that the regulated production of ROS is essential for fungal sexual development (Lara-Ortíz et al., 2003; Cano-Domínguez et al., 2008), a finding demonstrated in several fungi (Malagnac et al., 2004; Siegmund et al., 2013; Dirschnabel et al., 2014). In addition to their roles in the antioxidant response, AtfA and SrrA play different roles in gene regulation during development. AtfA mediates SakA roles in sexual development and conidial viability (Kawasaki et al., 2002; Lara-Rojas et al., 2011), while SrrA is required for normal conidiation and conidial viability (Vargas-Perez et al., 2007). The results reported here uncovered a novel developmental role for NapA, by showing that NapA represses sexual development and is needed for full conidiation, as well as for the accumulation of multiple mRNAs in conidia. The requirement for increased ROS levels during sexual development is consistent with the fact that Δ*napA* mutants show an increased production of fruiting bodies, as NapA is required to maintain low ROS levels and to express genes involved in the biosynthesis of cleistothecial melanin, a well-known antioxidant.

The lower conidiation observed in Δ*napA* mutants might be related to their lower expression of *gmcA* gene (AN8547), encoding a putative glucose-methanol-choline oxidoreductase required for early stages of conidiophore development (Etxebeste et al., 2012). The fact that GmcA ortholog AFUA_3G01580 is also induced by H_2_O_2_ in an AfYap1-dependent manner in *A. fumigatus* (Lessing et al., 2007) suggests that *gmcA* regulation by NapA is conserved, at least in the Aspergilli. Moreover, NapA roles in development might be conserved in fungi. In *M. oryzae* MoAP1 deletion causes only mild sensitivity to H_2_O_2_ but mutants show a drastic reduction in formation of aerial mycelium and conidiation. Notably, the same phenotypes are observed in mutants affected in MoAP1 regulated genes MGG_01230 and MGG_15157, encoding succinic semialdehyde dehydrogenase MoSsadh and acetyltransferase MoAct, respectively (Guo et al., 2011), which however we did not detect as NapA-dependent. In the dimorphic fungus *Talaromyces marneffei*, *yapA* mutants are sensitive to H_2_O_2_ and menadione, show decreased radial growth, produce conidiophores with fewer phialides and conidia, and conidia show decreased germination rates, while yeast cells fail to undergo binary fission (Dankai et al., 2016). Overall, the developmental roles of TFs long associated only with ROS detoxification support the role of ROS as developmental signals (Hansberg and Aguirre, 1990; Aguirre et al., 2005).

### Genes regulated by NapA during asexual development

A comparison between Ap1-like dependent regulons under H_2_O_2_ stress shows common themes in different fungi. DNA microarray analysis of *yap1*-dependent genes in *U. maydis* identified 221 down regulated genes with a fold change >1.5, that included genes for ROS decomposing enzymes and enzymes involved in biosynthesis of low molecular weight antioxidants and NADPH generation (Molina and Kahmann, 2007). Similarly, in *Cochliobulus heterostrophus* CHAP1-dependent genes included genes for thioredoxin reductase, γ-glutamyl cysteine synthetase, glutathione reductase, glutathione synthetase and thioredoxin (Lev et al., 2005). In *B. cinerea*, genes for catalaseC, thioredoxin reductase, glutaredoxin, glutathione-S-transferase1, thioredoxin and a hypothetical glutathione-S-transferase were found to be Bap1-dependent (Temme and Tudzynski, 2009) and a similar pattern was observed in *A*. *fumigatus* (Lessing et al., 2007).

Overall, such relatively conserved H_2_O_2_-induced Ap1-mediated gene expression patterns are different from the one we observe during conidiation. Indeed, we do not find genes involved in major NADPH or GSH generation pathways. Instead, we find a large number of genes involved in drug efflux and detoxification, including several genes for enzymes with putative cytochrome P450 activity, as well as other oxidases. Notably, Yap1 (Lee et al., 1999) and Pap1 (Chen et al., 2008) regulate genes coding for efflux pumps and dehydrogenases that seem necessary for defense against multiple drugs. In fact, *pap1* was first identified as a gene whose overexpression conferred resistance to drugs like brefeldin A, staurosporine or caffeine (Toda et al., 1991). Furthermore, Calvo et al. (2012) have shown that in response to H_2_O_2_ Pap1 requires the transcription factor Prr1 to activate the antioxidant but not the drug tolerance genes, providing a possible mechanism to explain the major role that NapA shows in the regulation of drug tolerance genes during conidiation. Indeed, the possibility that NapA plays overlapping but different functions during oxidative stress, conidiation and sexual development deserves further research.

Among the NapA-dependent genes, we also identified two members of the cupin superfamily, which includes metal-dependent and independent enzymes, as well as catalytically inactive proteins associated with abiotic stress and quiescent structures. AN8614 encodes a single-domain 153 amino-acid cupin showing similarity to germins and oxalate oxidases (Dunwell et al., 2000), some of which surprisingly also show superoxide dismutase activity (Woo et al., 2000). AN8368 encodes a 305 amino-acid protein identified as a spherulin 4-like protein. We detected that AN8368 is an ortholog of AN2952, and *A. fumigatus* and *A. clavatus* Sph3, which correspond to a glycoside hydrolase essential for the biosynthesis of the exopolysaccharide galactosaminogalactan (Bamford et al., 2015).

### The role of NapA in carbon catabolism

Conidia and mycelia from Δ*napA* mutants are unable to grow on ethanol and arabinose and show reduced growth on fructose, as sole carbon sources. A derepression of *alcA* during conidiation was observed before in conidiation defective mutants carrying alleles of the affected gene fused to the *alcA* promoter (Arratia-Quijada et al., 2012). The lack of growth on ethanol can be at least partially explained by the fact that NapA is required for full expression of *alcR, alcA*, and *aldA* genes during conidiation and presumably also during mycelial growth. Since NapA is needed for normal expression of AlcR and this TF is required for its own transcription, it seems likely that AlcR mediates the regulation of *alcP, alcA, alcM, alcS*, and *aldA* exerted by NapA. In this scenario *alcR* full expression might require CreA derepression, activation by NapA, as well as AlcR autoinduction. The fact that *alcR* promoter contains putative Ap1 binding sites is consistent with this interpretation. It seems unlikely that CreA represses *napA* expression because NapA antioxidant function must be required during normal glucose metabolism. In addition, *creA*^*d*^*204* mutants do not show increased resistance to H_2_O_2_ and qPCR analysis did not show increased *napA* mRNA levels in conidia from the *creA*^*d*^*204* mutant, as compared to wild type conidia (not shown). Three lines of evidence indicate that ethanol utilization represents a condition in which NapA antioxidant role becomes more important. First, NapA is required for full expression of most *alc* genes during conidiation. Second, ethanol growth induces *napA* expression and NapA nuclear localization. Third, NapA is needed for growth in ethanol. Evidence indicates that ethanol toxicity is mediated by AlcA product acetaldehyde, which itself can cause oxidative stress. Interestingly, it has been considered unlikely that ethanol catabolism alone requires such a high expression levels and subtle regulation of the *alc* genes (Flipphi et al., 2006). We have added another regulation layer that might help to understand the physiological significance of this complex regulation.

NapA requirement to utilize arabinose and fructose might be explained at different levels. First, NapA is necessary for transcript accumulation of the xylitol/sorbitol dehydrogenase (AN2666) in conidia and possibly in mycelia. AN2666 is 52% identical to *A. niger* enzyme XdhA, which participates in conversation of xylitol to D-xylulose during arabinose metabolism (de Groot et al., 2007). Xylitol/sorbitol dehydrogenase also shows 77% identity to SdhA, an enzyme involved in reversible transformation between fructose and sorbitol (Koivistoinen et al., 2012). This suggests that AN2666 participates in fructose assimilation and is essential for arabinose assimilation. In addition, arabinose and/or fructose transport might be compromised in Δ*napA* mutants, considering that NapA regulates several genes encoding proteins with transport activity. Among these, AN10891 and AN2665 encode proteins that are 28 and 26% identical to *B. cinerea* fructose specific transporter FRT1 (Doehlemann et al., 2005). Second, arabinose and fructose metabolism share some regulatory steps. Within the Aspergilli transcriptional activators AraR, GalR and XilR regulate the metabolic conversion of L-arabinose, D-galactose and D-xylose, respectively. A recent report shows that both AraR and XlnR regulate the pentose catabolism genes, as well as the oxido-reductive D-galactose catabolic pathway (Kowalczyk et al., 2015). However, it is interesting that mutants lacking both AraR and XlnR still show a detectable growth on arabinose, indicating the participation of additional regulatory mechanisms. Also interesting is that *A. niger* XlnR is somehow related to ROS metabolism, as *xlnR* null mutants are sensitive to oxidative stress and show a qualitative increase in ROS levels (Raulo et al., 2016). Third, NapA homologs are necessary for expression of enzymes involved in the generation of cellular reducing power (NADPH, GSH and thioredoxins). Although this was not detected during conidiation, during growth *A. nidulans* Δ*napA* mutants show a decreased GSH/GSSG ratio and a 1.6 fold decrease in total glutathione content when compared to a WT strain (Thön et al., 2010). This is consistent with the fact that Δ*napA* mutants are very sensitive to AA/acrolein, known to cause GSH depletion (Kwolek-Mirek et al., 2009). Such a decrease in reducing power might provide additional difficulties in catabolizing carbon sources that are more demanding on these resources. Indeed, ethanol and arabinose catabolism results in an imbalance of redox cofactors (Seiboth and Metz, 2011).

A role for NapA homologs in carbon metabolism appears conserved in fungi. In *S. cerevisiae* the involvement of Yap1 in responding to carbon stress was proposed after it was recovered in two-hybrid assays as interactor of the Sip2 subunit of the Snf1 kinase, needed for adaptation to glucose limitation, and showing that media shifting from glucose to glycerol or to glucose lacking media induced Yap1 nuclear accumulation (Wiatrowski and Carlson, 2003). Likewise, in *S. pombe* glucose but not nitrogen starvation induces Pap1 nuclear localization in a process that depends on the stress MAPK Spc1/Sty1 (Madrid et al., 2004). However, a direct involvement of Yap1 or NapA in carbon utilization was not demonstrated in these cases.

In nature, fungal spores germinate on environments containing diverse, often-poor carbon sources as well as drugs produced by competing organisms or that are detritus-derived. In addition, conidia contain chemicals and toxins derived form secondary metabolism as well as auto-inhibitors of germination. Our results support the proposal that oxidative stress is produced during conidiation and that NapA plays a crucial role in the regulation of multiple genes during this process. Although the rates of conidia germination are not decreased in Δ*napA* mutants under laboratory conditions, the set of genes regulated by NapA during conidiation suggest that they might be important for spores to germinate in natural heterogeneous environments.

## Author contributions

JA: Designed experiments, wrote the MS, and obtained funding. AM, FL and OS: Performed and designed experiments, contributed to MS writing.

### Conflict of interest statement

The authors declare that the research was conducted in the absence of any commercial or financial relationships that could be construed as a potential conflict of interest.
