# Supplementary material for: NapA Mediates a Redox Regulation of the Antioxidant Response, Carbon Utilization and Development in Aspergillus nidulans
Source: Front Microbiol. 2017 Mar 30;8:516. doi: 10.3389/fmicb.2017.00516 (PMC5371717; doi:10.3389/fmicb.2017.00516)
Supplement: Supplementary file 1 [file Table1.PDF]

Table S1. Stress signal transduction proteins in model fungi

Fungal phosphorelay components involved in stress sensing

Phosphorelay Component	*S. pombe*	*S. cerevisiae*	*A. nidulans*	*A. fumigatus*
**Sensor Kinase (HK)**	Mak1<br>Mak2<br>Mak3	Sln1*	15 HKs*	13 HKs*
**Phosphotransfer Protein (HPt)**	Mpr1	Ypd1	YpdA (AN2005)	Ypd1 (Afu4g10280)
**Response Regulator (MAPK-linked)**	Mcs4	Ssk1	SskA (AN7697)	Ssk1 (Afu5g08390)
**Response Regulator (TF)**	Prr1	Skn7	SrrA (AN3688)	Skn7 (Afu6g12520)

Fungal MAPK components involved in stress sensing

MAPK Component	*S. pombe*	*S. cerevisiae*	*A. nidulans*	*A. fumigatus*
**MAPKKK**	Wis4, Win1	Ssk2, Ssk22	SskB (AN10153)	SskB (Afu1g10940)
**MAPKK**	Wis1/Sty2	Pbs2	PbsA (AN0931) (Alias PbsB)	Pbs2 (Afu1g15950)
**MAPK**	Spc1/Sty1	Hog1	SakA/HogA (AN1017)<br>MpkC (AN4668)	SakA (Afu1g12940)<br>MpkC (Afu5g09100)
**bZIP TF**	Atf1	Sko1	AtfA (AN2911)	AtfA (Afu3g11330)

Fungal Ap1 transcription factors involved in oxidative stress sensing

AP1-like Component	*S. pombe*	*S. cerevisiae*	*A. nidulans*	*A. fumigatus*
**bZip TF**	Yap1	Pap1	NapA (AN7513)	AfYap1 (Afu6g09930)

* *S. pombe* HKs are involved in H_2O_2 sensing and *S. cerevisiae* Sln1 in osmotic stress sensing. HKs involved in oxidative stress sensing in *A. nidulans* and *A. fumigatus* have not been identified.
