# Supplementary material for: NapA Mediates a Redox Regulation of the Antioxidant Response, Carbon Utilization and Development in Aspergillus nidulans
Source: Front Microbiol. 2017 Mar 30;8:516. doi: 10.3389/fmicb.2017.00516 (PMC5371717; doi:10.3389/fmicb.2017.00516)
Supplement: Supplementary file 2 [file Table2.pdf]

Table S2. ***Aspergillus nidulans*** **strains used in this study**

Strain	Genotype	Source
FGSCA4	*biA1*	FGSC
CLK43	*pabaA1 yA2 veA1*	Kawasaki *et al*., 2002
A11035	*pyrG89 pyroA4 riboB2 ΔnkuA::argB veA1*	M. Hynes, Nayak *et al*., 2006
A1155	*pyrG89 pyroA4 ΔnkuA::bar veA1*	FGSC
CFL3	*pabaA1 yA2 pyrG89 veA1*	Lara-Rojas *et al*., 2011
CFL7	*pabaA1 yA2 ΔnapA::AfpyrG veA1*	This work, progeny from CFL3 X TFL9
$CreA^{d}204$	*pabaA1 yA2* $creA^{d}204$ *veA1*	P. González, progeny from CLK43 X MH440
MH440	*yA2 suA1adE20 adE20 riboB2 areA217* $creA^{d}204$	M. Hynes
TRN1	*pabaA1 yA2 ΔargB::trpCΔB: ΔcatA::argB trpC801 veA1*	Navarro *et al*., 1996
TLK12	*pabaA1 yA2 ΔargB::trpCΔB: ΔcatB::argB trpC801 veA1*	Kawasaki *et al*., 1997
TFL9	*pyrG89 pyroA4 riboB2 ΔnapA::AfpyrG ΔnkuA::argB veA1*	This work, 11035 transformed with PCR construct napA-AfpyrG-napA
TFL14	*pyrG89 pyroA4 Δ napA::AfpyrG Δ nkuA::argB veA1*	This work, 1155 transformed with PCR construct napA-gfp-AfpyrG
TFL15	*pyrG89 pyroA4 riboB2 Δ napA::AfpyrG Δ nkuA::argB veA1*	This work, 11035 transformed with PCR construct napA-AfpyrG-napA
TFLΔatfA-04	*pyrG89 pyroA4 riboB2 Δ atfA::AfriboB Δ nkuA::argB veA1*	Lara-Rojas *et al*., 2012
TΔsrrA-pyrG9	*pyrG89 pyroA4 riboB2 Δ srrA::AfpryG Δ nkuA::argB veA1*	Vargas-Pérez *et al*., 2007
TAM16	*pyrG89 pyroA4 riboB2 Δ gpxA::AfriboB Δ nkuA::argB veA1*	This work, 11035 transformed with PCR construct gpxA-AfriboB-gpxA
TAM17	*pyrG89 pyroA4 riboB2 Δ tpxA::AfpyrG Δ nkuA::argB veA1*	This work, 11035 transformed with PCR construct tpxA-AfpyrG-tpxA
TAM19	*pyrG89 pyroA4 riboB2 Δ tpxB::AfRiboB ΔnkuA::argB veA1*	This work, 11035 transformed with PCR construct tpxB-AfriboB-tpxB
TAM20	*pyrG89 pyroA4 riboB2 Δ alcA::AfRiboB ΔnkuA::argB veA1*	This work, 11035 transformed with PCR construct alcA-AfriboB-alcA
COS SrrA3.1	*pabaA1 yA2 Δ srrA::AfpyrG veA1*	This work, progeny from TSrrAΔsskA X CLK56
CAM6	*pabaA1 yA2 Δ napA::AfpryG Δ srrA::AfpryG veA1*	This work, progeny from CFL7 X TΔsrrA-pyrG9
CAM7	*pabaA1 yA2 Δ napA::AfpryG Δ atfA::AfriboB veA1*	This work, progeny from ΔatfA-04 X CAM6
CAM8	*pabaA1 yA2 Δ srrA::AfpryG Δ atfA::AfriboB veA1*	This work, progeny from ΔatfA-04 X CAM6
CAM9	*pabaA1 yA2 Δ srrA::AfpryG Δ napA::AfpyrG Δ atfA::AfriboB veA1*	This work, progeny from ΔatfA-04 X CAM6
CAM11	*pabaA1 yA2 Δ gpxA::AfriboB veA1*	This work, progeny from TAM16 X CLK43

CAM12	*pabaA1, yA2 ΔgpxA::AfriboB Δtpx A::AfpyrG veA1*	This work, progeny from CAM11 X TAM17
CAM13	*pabaA1 yA2 ΔtpxA::AfpyrG veA1*	This work, progeny from CAM11 X TAM17
CAM14	*pabaA1 yA2 ΔgpxA::AfriboB ΔtpxA::AfpyrG ΔnapA::AfpyrG veA1*	This work, progeny from CAM12 X TFL9
CAM15	*pabaA1 yA2 ΔgpxA::AfriboB ΔtpxB::AfriboB ΔtpxB::AfpyrG veA1*	This work, progeny from CAM12 X TAM19
CAM16	*pabaA1 yA2 ΔgpxA::AfriboB ΔtpxA::AfpyrG ΔtpxB::AfriboB ΔnapA::AfpyrG veA1*	This work, progeny from CAM14 X TAM19
CAM17	*pabaA1 yA2 ΔalcA::AfriboB veA1*	This work, progeny from TAM20 X 11035
CAM18	*pabaA1 yA2 ΔalcA::AfriboB ΔnapA::AfpyrG veA1*	This work, progeny from CAM17 X TFL9
COS440	*pabaA1 yA2 ΔnapA::AfpyrG*	This work, progeny from CFL7 X FGSCA4
COS441	*pabaA1 yA2 ΔnapA::AfpyrG*	This work, progeny from CFL7 X FGSCA4
