# Supplementary material for: NapA Mediates a Redox Regulation of the Antioxidant Response, Carbon Utilization and Development in Aspergillus nidulans
Source: Front Microbiol. 2017 Mar 30;8:516. doi: 10.3389/fmicb.2017.00516 (PMC5371717; doi:10.3389/fmicb.2017.00516)
Supplement: Supplementary file 3 [file Table3.PDF]

Table S3. DNA primers used in this study

Primer	Sequence (5’ to 3’)
5’For-napA	CGATGTGAGAAAGGCACG
5’Rev-napA	GGTGAAGAGCATTGTTTGAGGCCGGTCCTGGAAGAGAGG
3’For-napA	GCATCAGTGCCTCCTCTCAGACCACGCTACGGATACCCAC
3’Rev-napA	GCGGTAGCAACACATCAC
5’nest-napA	CTGCACGCGTGCTGGTCG
3’nest-napA	TCGTCCGCTCCATCAACG
5Dig-nkuA	GATGCTGTCCTTTTTGCC
3Dig-nkuA	TACCCCAGAGCGCGATAC
pyrG forward	GCCTCAAACAATGCTCTTCACC
pyrG reverse	GTCTGAGAGGAGGCACTGATGC
5Ribo	CTGGCTCGTTTGATCACATGG
6Ribo	GCGCTGCAGAACCGTTACATG
GFP1napA	AGGGATGTGGATGACATCATTGGCCGCGTGGGAGCTGGTGCAGGCGCTGGAGCCGGTGCC
GFP2napA	CAGACTCTCCTCCGTGACTACGTTGGTCAAGCGAATCGAATTGAACAATCGA
GSP1napA	CTGGCACTGCAATTTGGTCTGGCCGTCC
GSP2napA	CACGCGGCCAATGACGTCATCCACATCCCT
GSP3napA	TCGATTGTTCAATTCGATTCGCTTGACCAA
GSP4napA	TACGTCTCCTGCTCCCGTCATCCCTAAG
5’AtfFor	ATACCGCTACGAATCCAGACCC
5´For-gpxA	CCATAAAAACCGTGGTAG CTCAGGA
5´Rev-gpxA	CGAGCCAGACTCCTGAACGGCCTCTTAGGTCTGAGATGAGGTTGTTGACGTT
3´For-gpxA	AACGGTTCTGCAGCGCAAACGTTCTTTTTTACATTGTTATTTCAAGGATTA
3´Rev-gpxA	CGGAACCGGTAACAACAATAAGTTG
5´For-nested gpxA	CGGAAGCCGATCTCCCCT
3´For-nested gpxA	GCAGTGTATGTTATCTCTGACCCAAGG
5´For-tpxA	CGATTCGGCAAGATATCAAA
5´Rev-tpxA	GAAGAGCATTGTTTGAGGCTGTGAGAGTGTTGATGTATCAGAA
3´For-tpxA	AGTGCCTCCTCTCAGACATTGGAAAATGATACCTCATAACC
3´Rev-gpxA	CGACTGGCTTCTCGACATC
5´For-nested tpxA	TAGCACACCGGGTTCAGAG
3´For-nested tpxA	TCGTGAGGTGTAGATACGGTGT
5´For-tpxB	GATACCATGCATTATTAGTA
5´Rev-tpxB	TGATCAAACGAGCCAGTTTGATATTTTCACAGCA
3´For-tpxB	TAACGGTTCTGCAGCGCTGTAATCTGAATAGAATGTACTAT
3´Rev-tpxB	GGTTGATCTTCATTCTCTC
5´For-nested tpxB	TTTCAAAGCACTATGATGAT
3´For-nested tpxB	TTTTTCTTTCAATCGCT
5´For-alcA	AATCACAACACCACGGTCTGCTAC
5´Rev-alcA	CCATGTGATCAAACGAGCCAGTTTGAGGCGAGGTGATAGGATTG
3´For-alcA	CATGTAACGGTTCTGCAGCGCGCGTTTCAACGGCCACGG

3´Rev-alcA	AGACCCAGAAGCTATGGGGTACTTG
5´For nested-alcA	CAACGGGCGAGAGGAGCA
3´Rev nested-alcA	GTAGGATGATAGGCGACCGACATA
alcA for	GGTCGATGGGGGAGATGAGAAG
alcA rev	CGTGCGCACCGAGACCAT
AldA for	GGTCCTGTCGTCACTATCCAGAAGTTC
AldA rev	ACACGCGAATGGCGGTGTT
AlcR for	GGCCCTGCACCTACGAACAG
AlcR rev	CGCTTCAAGGCGGGCAG
NapA for	ATGGATAACAAGGAGGAGGAAGAGG
NapA rev	GATTGTCAACGTCGATTTCACCG
