# Supplementary material for: NapA Mediates a Redox Regulation of the Antioxidant Response, Carbon Utilization and Development in Aspergillus nidulans
Source: Front Microbiol. 2017 Mar 30;8:516. doi: 10.3389/fmicb.2017.00516 (PMC5371717; doi:10.3389/fmicb.2017.00516)
Supplement: Supplementary file 4 [file Table4.PDF]

Table S4. Down regulated transcripts in Δ*napA* conidia with LogFC values between 2.0 and 1.5

Gene	LogFC	Description and Domains	Gene	LogFC	Description and Domains
AN7858	1.99		AN7972	1.71	
AN6462	1.99		AN12193	1.70	
AN1643	1.98		AN5967	1.70	
AN7279	1.98	Has domain(s) with predicted poly(beta-D-mannuronate) lyase activity, role in alginic acid catabolic process and periplasmic space localization	AN2344	1.70	
AN4829	1.98	Putative reductase with a predicted role in carbohydrate metabolism	AN5416	1.70	
AN2667	1.97	Ortholog(s) have role in conidiophore development	AN7081	1.70	
AN2716	1.97	Has domain(s) with predicted catalytic activity and role in metabolic process	AN0585	1.69	
AN2777	1.97	Has domain(s) with predicted catalytic activity and role in metabolic process	AN1568	1.69	
AN9288	1.97		AN2675	1.69	
AN8512	1.96		AN9320	1.69	
AN3399	1.96		AN12291	1.68	
AN12024	1.96		AN0841	1.68	
AN7901	1.95		AN3529	1.68	
AN8978	1.95	alcR	AN12268	1.67	
AN12218	1.94		AN1271	1.67	
AN8548	1.94		AN2503	1.67	
AN7113	1.94		AN4061	1.67	
AN0607	1.93	Non-ribosomal peptide synthetase; similar to ferrichrome peptide synthetases; involved in ferricrocin (FC) siderophore biosynthesis	AN3570	1.67	
AN8953	1.92	Putative alpha-glucosidase with a predicted role in maltose metabolism; transcriptionally induced by isomaltose; induced by rapamycin-induced autophagy	AN1302	1.66	
AN11807	1.92		AN8752	1.66	
AN1649	1.91		AN8525	1.66	
AN12381	1.91		AN4367	1.66	Putative chitin synthase III with a predicted role in chitin biosynthesis; transcript is induced by nitrate
AN5672	1.91		AN8779	1.66	
AN8390	1.90		AN8345	1.66	
AN8441	1.90		AN7199	1.66	
AN5415	1.90		AN7505	1.65	Protein with alpha-xylosidase activity, involved in degradation of xyloglucans
AN9009	1.90		AN8493	1.65	
AN7269	1.90		AN8697	1.65	
AN2778	1.90		AN1868	1.65	
AN8138	1.89	Alpha-galactosidase, involved in degradation of mannans; predicted role in galactose and galactitol metabolism; glycoside hydrolase family 36 (GH36); transcriptionally induced by growth on xylose	AN0172	1.65	

AN2702	1.89		AN8027	1.64	
AN7619	1.89		AN0389	1.64	
AN3244	1.89		AN7131	1.64	*CYP52H1*
AN11043	1.89		AN3066	1.64	
AN6808	1.89		AN2361	1.64	
AN2664	1.88		AN3099	1.64	
AN9013	1.88		AN7265	1.64	
AN3638	1.88		AN1703	1.64	*CYP5128A1*
AN3590	1.88		AN5905	1.64	
AN1822	1.88		AN11702	1.64	
AN8532	1.87		AN1640	1.64	
AN5860	1.87	Low affinity glucose transporter of the major facilitator superfamily (MFS); transcriptionally repressed by growth on xylose	AN6133	1.63	
AN2989	1.87		AN6293	1.63	
AN8008	1.87		AN11094	1.63	
AN8347	1.86		AN9263	1.62	
AN1785	1.86		AN3631	1.62	
AN3765	1.85		AN7958	1.62	
AN1737	1.85	*CYP567C1*	AN1566	1.62	
AN1182	1.85	benA; Beta-tubulin, highly conserved component of microtubules; A. nidulans has two beta-tubulin genes, benA and tubC; temperature sensitive mutants are blocked in mitosis and in nuclear division	AN2204	1.62	
AN8249	1.85		AN4806	1.62	
AN11950	1.85		AN3304	1.62	
AN10909	1.85		AN11212	1.61	
AN8982	1.85	Predicted protein of unknown function; member of the alc gene cluster; alcU	AN11759	1.61	
AN6649	1.85	Putative fatty acyl-CoA synthetase	AN2862	1.61	
AN7806	1.85	Putative versicolorin reductase with a predicted role in sterigmatocystin/aflatoxin biosynthesis; member of the sterigmatocystin biosynthesis gene cluste	AN8406	1.61	
AN8983	1.84		AN3195	1.61	
AN4135	1.84	Putative delta-9-stearic acid desaturase; converts palmitic acid and stearic acid to palmitoleic acid and oleic acid; null mutant has decreased fatty acid content; synthetically lethal with sdeA mutation	AN8610	1.61	
AN8615	1.84	*CYP677A1*	AN2717	1.61	Putative RNA-directed RNA polymerase
AN6128	1.84		AN6748	1.61	Putative pectate lyase with a predicted role in pectin degradation
AN3487	1.83		AN10815	1.60	
AN12402	1.83	Prenyltransferase; deletion leads to the elimination of the prenyl xanthones, shamixanthone and epishamixanthone, and the accumulation of several metabolites including a monodictyphenone derivative	AN5716	1.60	
AN7691	1.83	Putative phospholipase	AN6941	1.60	
AN9139	1.82		AN3497	1.60	*CYP623B2*
AN5252	1.82		AN2674	1.60	

AN7510	1.82		AN7268	1.60	
AN6729	1.81		AN7128	1.60	
AN11034	1.81		AN3482	1.60	
AN0146	1.81	Protein with homology to versicolorin ketoreductase; member of the (mdp) monodictyphenone secondary metabolite biosynthesis gene cluster; required for monodictyphenone biosynthesis	AN4355	1.60	
AN8298	1.81		AN11797	1.60	
AN2644	1.81		AN9425	1.60	
AN7264	1.81		AN6421	1.59	
AN7637	1.81		AN6400	1.59	
AN1218	1.81		AN4859	1.59	Plasma membrane ATPase with a predicted role in energy metabolism
AN6234	1.81		AN11196	1.59	
AN0765	1.81		AN1197	1.59	
AN0050	1.81		AN8504	1.59	
AN10972	1.81		AN3979	1.58	
AN11819	1.80		AN1202	1.58	Conserved nudix hydrolase isozyme
AN10575	1.80		AN4026	1.58	
AN4893	1.80		AN0698	1.58	
AN3774	1.80		AN10886	1.58	
AN8492	1.80		AN5913	1.58	
AN5053	1.80		AN4792	1.58	
AN1839	1.80		AN9235	1.58	
AN2186	1.80		AN7583	1.58	
AN7917	1.79		AN7242	1.58	
AN6871	1.79		AN0019	1.58	
AN2343	1.79		AN9308	1.57	
AN1204	1.79		AN8971	1.57	
AN6167	1.79		AN3269	1.57	
AN11080	1.79		AN5283	1.57	
AN6369	1.78		AN9164	1.56	
AN3215	1.78		AN5859	1.56	
AN7124	1.78		AN3903	1.55	Putative beta-glucosidase with a predicted role in polysaccharide degradation
AN7667	1.78		AN5988	1.55	
AN2598	1.78		AN1681	1.55	
AN7267	1.77		AN11948	1.55	
AN7707	1.77		AN6744	1.55	
AN5509	1.77		AN10049	1.55	Protein with homology to scytalone dehydratase; member of the monodictyphenone (mdp) secondary metabolite biosynthesis gene cluster; transcript is induced by nitrate
AN8476	1.77		AN7952	1.54	
AN0830	1.77		AN6075	1.54	
AN10898	1.77		AN5061	1.54	Putative xyloglucanase
AN9181	1.77		AN7631	1.54	

AN4795	1.76		AN8123	1.54	Putative fructosyl amino acid oxidase, catalyzes oxidative deglycation of glycated amines (Amadori compounds); induction of expression by fructosyl amines depends in veA gene
AN1072	1.76		AN4989	1.54	
AN5431	1.76		AN3414	1.54	
AN8777	1.76		AN6883	1.53	
AN2622	1.76	Isopenicillin-N synthase with a role in penicillin biosynthesis; expression is negatively regulated by glucose and acidic pH	AN4773	1.53	
AN4999	1.75		AN10623	1.53	
AN4134	1.75		AN11711	1.53	
AN8479	1.75		AN11046	1.53	
AN5407	1.75		AN7927	1.53	
AN4507	1.74	Putative alpha-amylase with a predicted role in starch metabolism; predicted glycosyl phosphatidylinositol (GPI)-anchor	AN6647	1.53	
AN4006	1.74		AN10384	1.53	
AN5930	1.74		AN6775	1.53	
AN10500	1.74		AN0750	1.53	
AN1866	1.74		AN8222	1.53	
AN7200	1.74		AN12243	1.53	
AN3278	1.74		AN4124	1.52	
AN2869	1.74		AN5674	1.52	MAP kinase, kinase, kinase, kinase (MAPKKKK); mutants undergo premature but incomplete sexual development
AN3895	1.74		AN3499	1.52	
AN3100	1.74		AN2016	1.52	
AN10571	1.73		AN7266	1.52	
AN5850	1.73		AN7068	1.52	
AN2994	1.73		AN9531	1.52	
AN9152	1.73		AN11267	1.52	
AN2668	1.73		AN2718	1.52	
AN9033	1.72		AN2590	1.52	
AN5055	1.72		AN0970	1.51	
AN9474	1.72		AN8467	1.51	
AN5434	1.72		AN6745	1.51	
AN7780	1.72		AN10319	1.51	
AN2864	1.72		AN6015	1.51	
AN4392	1.71		AN1797	1.51	Ortholog(s) have fructose transmembrane transporter activity, glucose transmembrane transporter activity, mannose transmembrane transporter activity
AN1477	1.71		AN4120	1.51	
AN1853	1.71		AN6940	1.50	
AN0638	1.71		AN0656	1.50	
AN5033	1.71		AN0147	1.50	Flavin-containing monooxygenase; member of the monodictyphenone secondary metabolite biosynthesis gene cluster; responsible for conversion of monodictyphenone to the prenyl xanthones, shamixanthone and epishamixanthone

AN6413	1.71	AN0902	1.50
AN7953	1.71	AN0738	1.50
AN2585	1.71	AN2592	1.50
AN11076	1.71	AN3299	1.50
