# Supplementary material for: NapA Mediates a Redox Regulation of the Antioxidant Response, Carbon Utilization and Development in Aspergillus nidulans
Source: Front Microbiol. 2017 Mar 30;8:516. doi: 10.3389/fmicb.2017.00516 (PMC5371717; doi:10.3389/fmicb.2017.00516)
Supplement: Supplementary file 5 [file Image1.PDF]

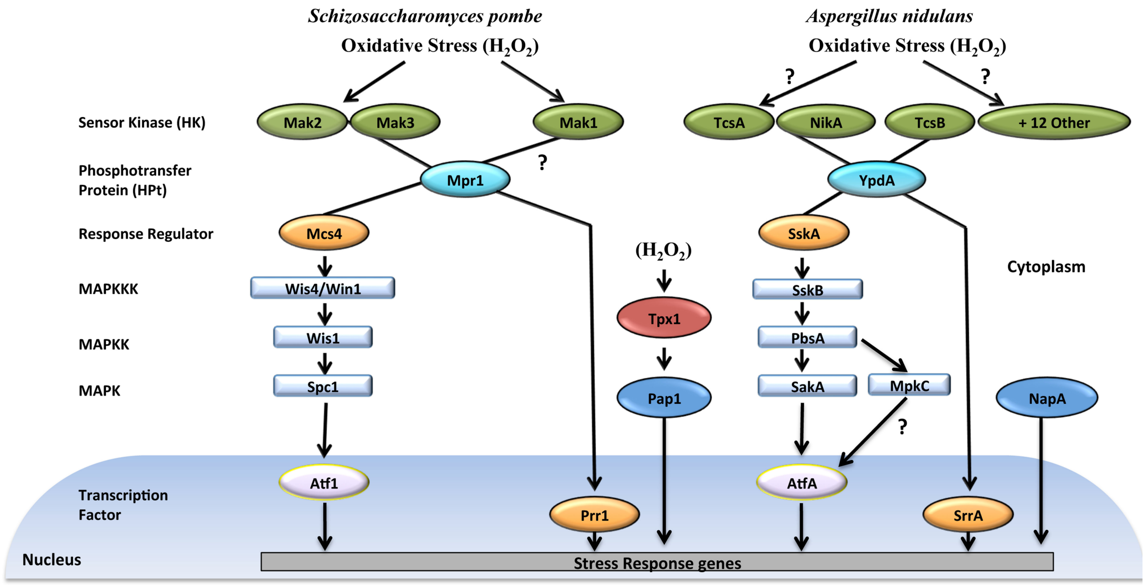

FIGURE S1. Oxidative stress sensing pathways in *S. pombe* and *A. nidulans*. The phosphorelay system composed by histidine kinases (green), phosphotransfer proteins (blue) and response regulators Mcs4 and SskA (orange) are connected to the stress MAPK module (light blue). When phosphorylated, Spc1 and SakA MAPKs translocate to the nucleus, where they activate bZIP transcription factors Atf1 and AtfA, respectively. Response regulators Prr1 and SrrA are located in the nucleus where they are activated by phosphotransfer proteins. In *S. pombe* bZIP transcription factor Pap1 oxidation by peroxiredoxin Tpx1 results in its nuclear localization and activation. HKs and peroxiredoxins responsible for H_2O_2 sensing have not been identified in *A. nidulans*. A direct interaction between MpkC and AtfA has not been demonstrated. See the text for detailed description and relevant references.
