# Supplementary material for: NapA Mediates a Redox Regulation of the Antioxidant Response, Carbon Utilization and Development in Aspergillus nidulans
Source: Front Microbiol. 2017 Mar 30;8:516. doi: 10.3389/fmicb.2017.00516 (PMC5371717; doi:10.3389/fmicb.2017.00516)
Supplement: Supplementary file 6 [file Image2.PDF]

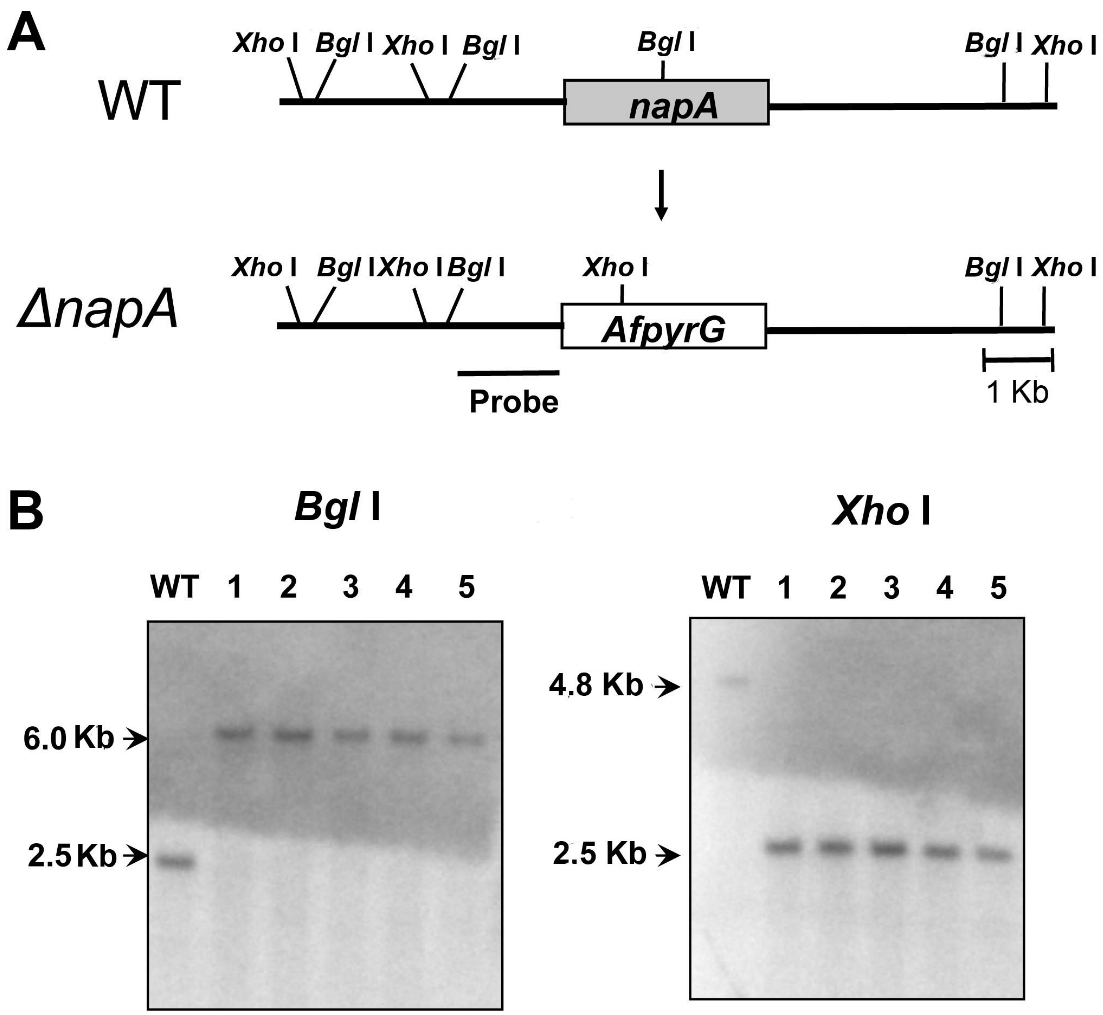

FIGURE S2. Deletion of the *napA* gene. **(A)** A *napA* deletion construct containing the A*fpyrG* gene, as a selective marker, was generated by double-joint PCR and used to transform strain 11035. The expected integration event results in replacement of the wild-type *napA* gene by the deletion construct. **(B)** DNA from strains 11035 (wild type; WT) and 5 $PyrG^{+}$ transformants were digested with BglI or XhoI and used for Southern blot analysis using the probe in indicated **(A)**. The wild type pattern corresponds a 2.5 Kb band for BglI and 4.8 Kb band for XhoI while *ΔnapA* pattern corresponds to bands of 6 and 2.5 Kb. Transformant 4 was named TFL9 and used in further experiments.
