# Supplementary material for: NapA Mediates a Redox Regulation of the Antioxidant Response, Carbon Utilization and Development in Aspergillus nidulans
Source: Front Microbiol. 2017 Mar 30;8:516. doi: 10.3389/fmicb.2017.00516 (PMC5371717; doi:10.3389/fmicb.2017.00516)
Supplement: Supplementary file 7 [file Image3.PDF]

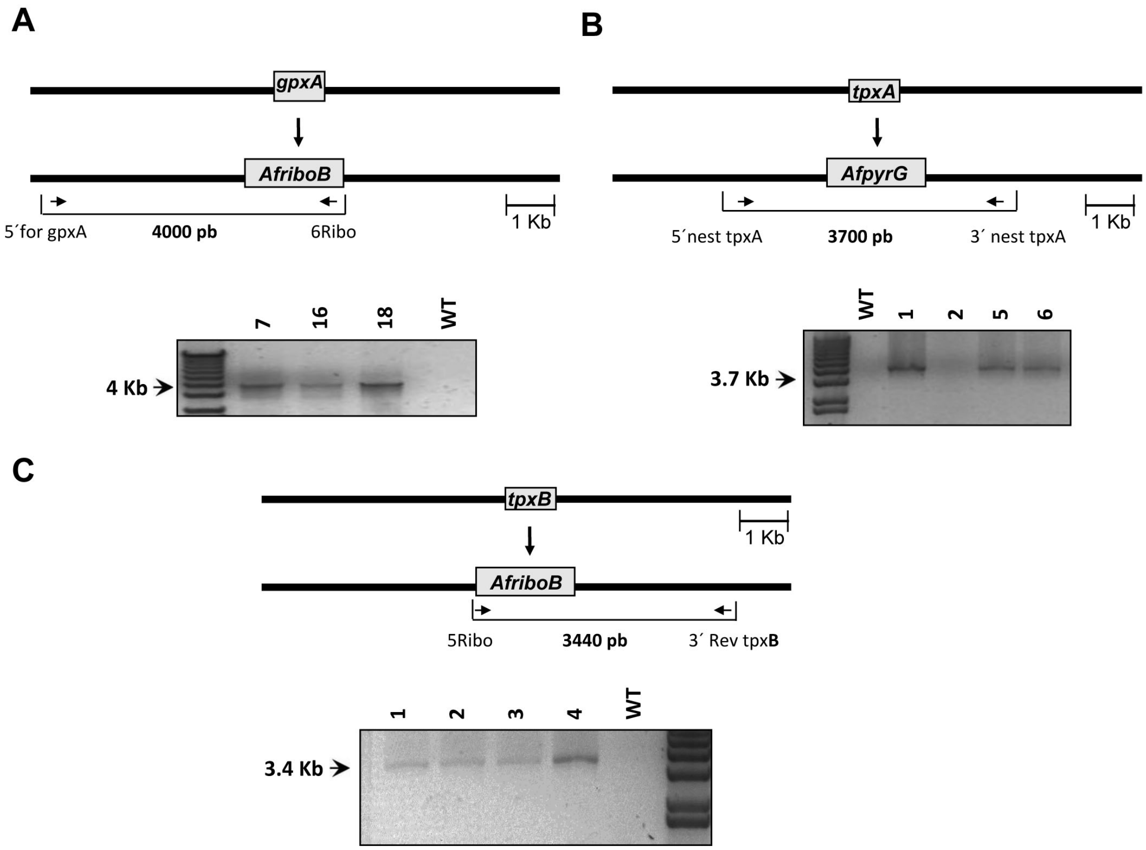

FIGURE S3. PCR analysis of Δ*gpxA*, Δ*tpxA* and Δ*tpxB* deletion strains. (A) DNA from WT (CLK43) and indicated $PyrG^+$ transformantswas used for PCR analysis. For *gpxA* gene deletion, primers 5´for gpxA and 6Ribo were utilized to amplify a 4 Kb band present only in *gpxA* deletion transformants. Transformant 18 was used for further experiments. **(B)** For *tpxA* gene deletion, 5´nest tpxA and 3´nest tpxA primers were used to amplifiy a 3.7 Kb band present only in *tpxA* deletion transformants. mants. Transformant 6 was used for further experiments. **(C)** For *tpxB* gene deletion, a 3.4 Kb band was obtained with primers 5Ribo and 3´ Rev tpxB only in transformants carrying a *tpxB* gene deletion. Transformant 4 was used for further experiments.
