# Supplementary material for: NapA Mediates a Redox Regulation of the Antioxidant Response, Carbon Utilization and Development in Aspergillus nidulans
Source: Front Microbiol. 2017 Mar 30;8:516. doi: 10.3389/fmicb.2017.00516 (PMC5371717; doi:10.3389/fmicb.2017.00516)
Supplement: Supplementary file 8 [file Image4.PDF]

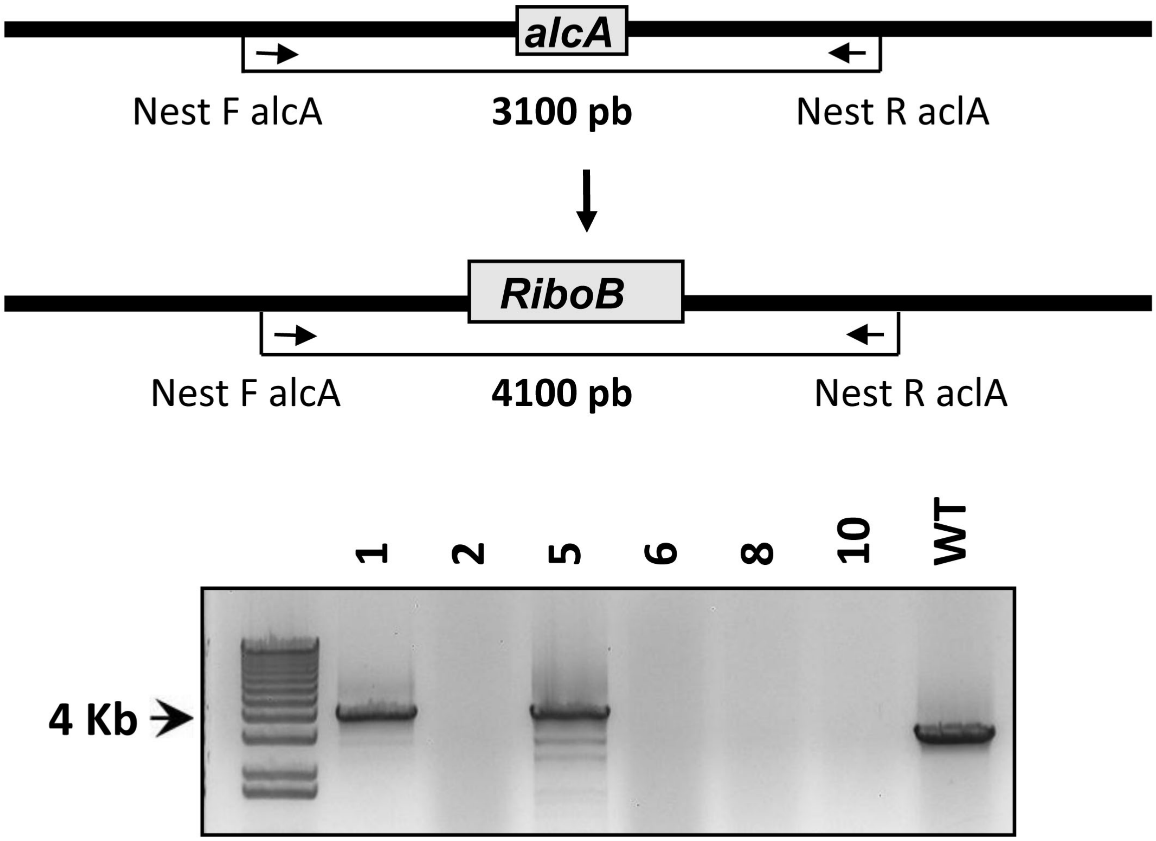

FIGURE S4. PCR analysis of Δ*alcA* deletion strains. *alcA* deletion construct containing *AfriboB* gene as a selective marker was generated by double-joint PCR and used to transform strain 11035. Primers NestFalcA and NestRaclA generated 4.1 and 3.1 Kb bands in *alcA* deletion and wild type strains, respectively. Transformant 1 was used for further experiments.
