# Supplementary material for: NapA Mediates a Redox Regulation of the Antioxidant Response, Carbon Utilization and Development in Aspergillus nidulans
Source: Front Microbiol. 2017 Mar 30;8:516. doi: 10.3389/fmicb.2017.00516 (PMC5371717; doi:10.3389/fmicb.2017.00516)
Supplement: Supplementary file 9 [file Image5.pdf]

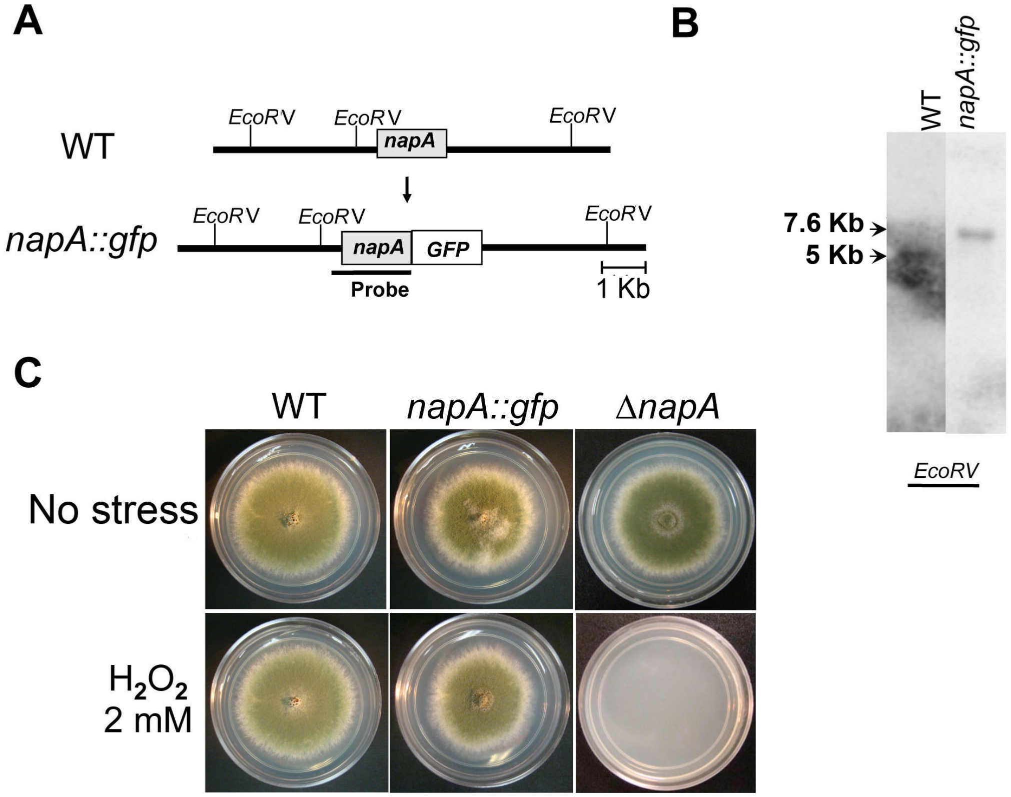

FIGURE S5. GFP tagging does not affect NapA function. **(A)** Three PCR products were used to generate an AtfA C-terminal GFP construct by double joint PCR, which was used to transform strain A1155. **(B)** Total DNA from strains A1155 (WT) and $PyrG^{+}$ transformant TFL14 were digested with EcoRV restriction enzyme and used for Southern blot analysis using *napA* ORF as probe. The WT pattern corresponds to a 5 Kb band, while *napA::GFP* pattern corresponds to a 7.6 Kb fragment. H_2O_2 resistance of WT and *napA::GFP* strains is similar. **(C)** Conidiospores ($1X10^4$) from strains A1155 (WT), TFL9 (Δ*napA*) and TFL14 (*napA::GFP*) were inoculated on supplemented MM plates with or without 2 mM H_2O_2 and incubated at 37°C for 4 days.
