# Supplementary material for: NapA Mediates a Redox Regulation of the Antioxidant Response, Carbon Utilization and Development in Aspergillus nidulans
Source: Front Microbiol. 2017 Mar 30;8:516. doi: 10.3389/fmicb.2017.00516 (PMC5371717; doi:10.3389/fmicb.2017.00516)
Supplement: Supplementary file 10 [file Image6.PDF]

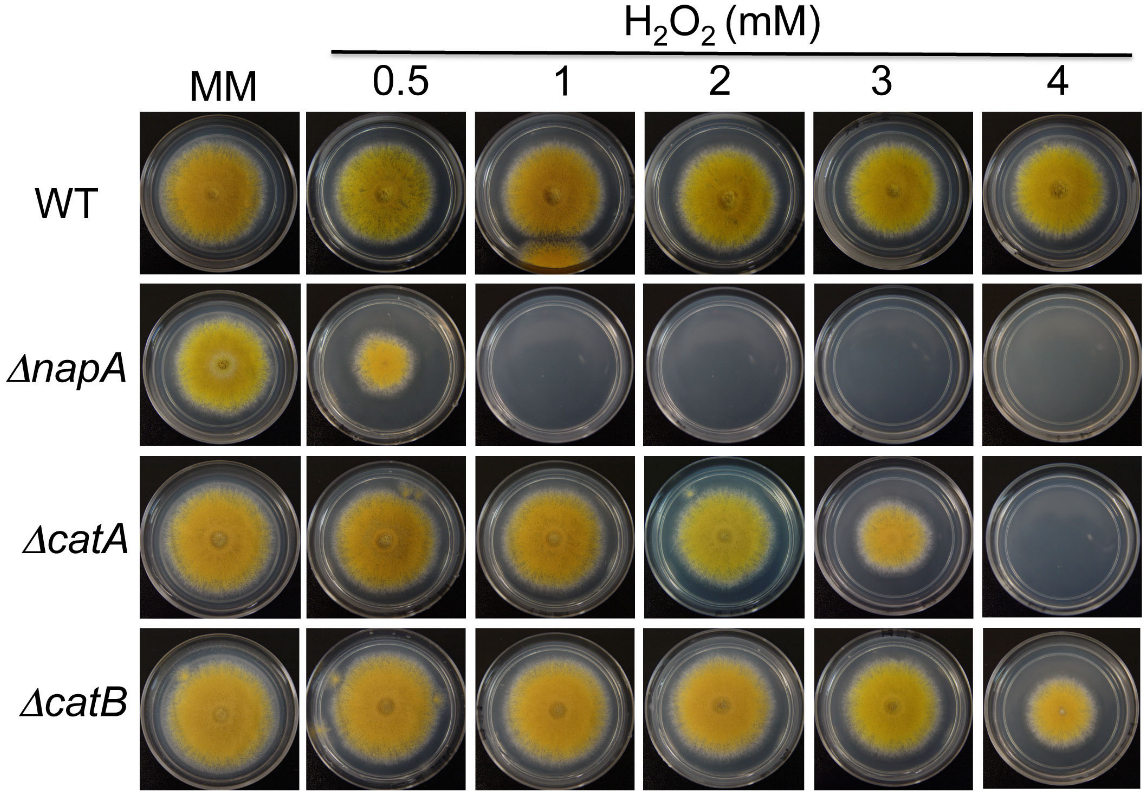

FIGURE S6. Δ*napA* mutants are more sensitive to H_2O_2 than catalase mutants Δ*catA* and Δ*catB*. Conidia ($1X10^4$) from strains CLK43 (WT), CFL7 (Δ*napA*), CRN1 (Δ*catA*) and TLK12 (*ΔcatB*) were inoculated on supplemented MM plates containing H_2O_2 at the indicated concentrations and incubated at 37°C for 4 days.
