# Supplementary material for: NapA Mediates a Redox Regulation of the Antioxidant Response, Carbon Utilization and Development in Aspergillus nidulans
Source: Front Microbiol. 2017 Mar 30;8:516. doi: 10.3389/fmicb.2017.00516 (PMC5371717; doi:10.3389/fmicb.2017.00516)
Supplement: Supplementary file 11 [file Image7.PDF]

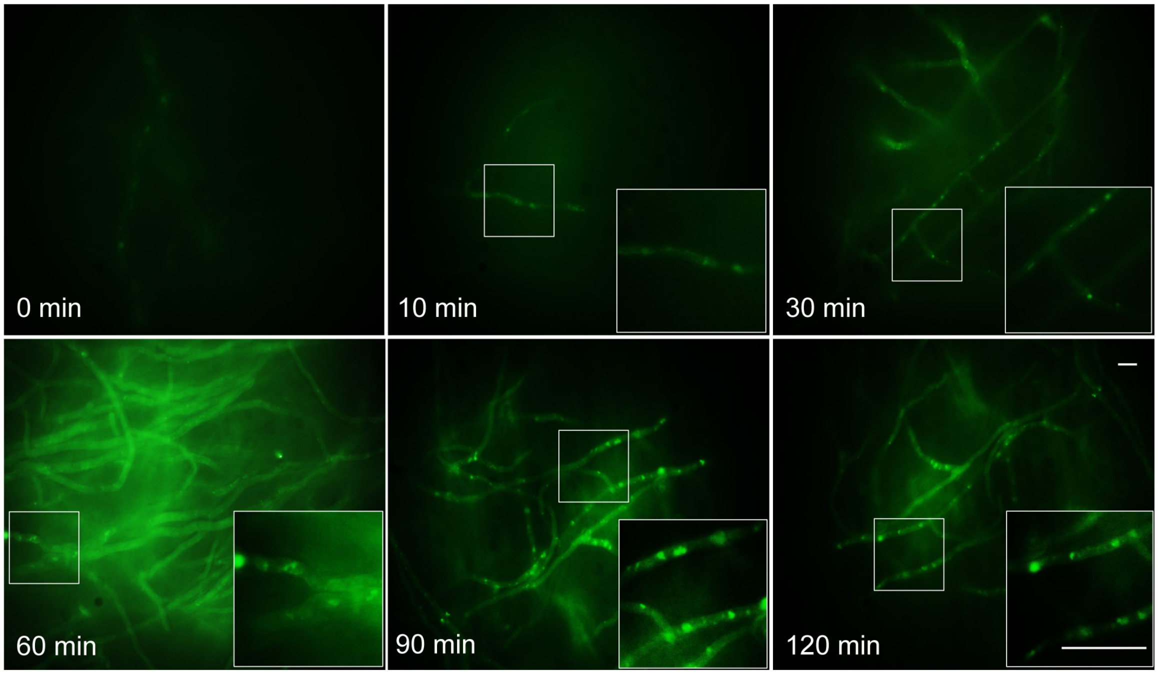

FIGURE S7. Osmotic stress induces NapA nuclear localization. Conidia from strain CAM20 (NapA::GFP) were grown for 18 h in liquid MM medium, which was then replaced by the same medium containing 1M NaCl for the indicated times (0-120 min). Mycelial samples were observed *in vivo* and photographed every 30 minutes using Epifluorescence microscopy. Bar = 10 µm.
